# First theoretical framework for highly efficient photovoltaic parameters by structural modification with benzothiophene-incorporated acceptors in dithiophene based chromophores

**DOI:** 10.1038/s41598-022-24087-8

**Published:** 2022-11-23

**Authors:** Muhammad Khalid, Rameez Ahmed, Iqra shafiq, Muhammad Arshad, Muhammad Adnan Asghar, Khurram Shahzad Munawar, Muhammad Imran, Ataualpa A. C. Braga

**Affiliations:** 1 Institute of Chemistry, Khwaja Fareed University of Engineering & Information Technology, Rahim Yar Khan, 64200 Pakistan; 2 Centre for Theoretical and Computational Research, Khwaja Fareed University of Engineering & Information Technology, Rahim Yar Khan, 64200 Pakistan; 3 Department of Chemical Engineering, College of Engineering, King Khalid University, Abha, Saudi Arabia; 4 Division of Science and Technology, Department of Chemistry, University of Education, Lahore, Pakistan; 5 Department of Chemistry, University of Mianwali, Mianwali, 42200 Pakistan; 6 Department of Chemistry, Faculty of Science, King Khalid University, P.O. Box 9004, Abha, 61413 Saudi Arabia; 7 Departamento de Química Fundamental, Instituto de Química, Universidade de São Paulo, Av. Prof. Lineu Prestes, 748, São Paulo, 05508-000 Brazil

**Keywords:** Chemistry, Optics and photonics

## Abstract

Now a days, researchers are constantly doing efforts to upgrade the performance of solar based devices with the aim of increasing the role of photovoltaic materials in modern hi-tech optoelectronic applications. Realizing the recent energy conditions across the globe, research is diverted from fullerene to non-fullerene electron acceptor moieties in this era, considering their remarkable contribution in organic solar cells (OSCs). Therefore, we designed seven novel non-fullerene fused ring electron acceptor chromophores (**MD2**–**MD8**) from **DOC2C6-2F** by structural tailoring with different acceptors at end-capped units. DFT study was performed at B3LYP functional to discover the opto-electronic characteristics of the newly tailored chromophores. Various analysis such as frontier molecular orbitals (FMOs), transition density matrix (TDM), density of states (DOS), binding energy (E_b_), reorganization energy, open circuit voltage (*Voc*) was carried out to comprehend the photovoltaic response of **MD2**–**MD8**. Decrease in band gaps (1.940–1.571 eV) with wider absorption spectrum (725.690–939.844 nm in chloroform) along with greater charge transfer rate from HOMO towards LUMO were examined in derivatives as compared to **MR1** (E_gap_ = 1.976 eV, λ_max_ = 738.221 nm) except **MD7**. Further, in all derivatives, smaller values of E_b_ (0.252–0.279 eV) were examined than that of reference (0.296 eV). These lower binding energy values of **MD2**–**MD8** indicated the higher rate of excitation dissociation with lager charger transfer rate than **MR1,** which further supported by DOS and TDM analyses. Additionally, least reorganization energy in the aforesaid compounds for hole with electron was also inspected. Moreover, *V*_*oc*_ a good photovoltaic response was noted for all studied compounds which indicated that these compounds are suitable to synthesize OSCs in future.

## Introduction

The technologies of OSCs have progressed in terms of architecture, processing techniques and the semiconductor materials^1,2^. The solar cells having a promising future as a sustainable and clean replacement of fossil fuel are organic solar cells (OSCs). Due to profound advantages in manufacturing, low weight, flexibility and less expensive this photovoltaic technology has been the attention of industrial and academic community for decades^3^. In current scenario, the most promising approach of converting sunlight into electrical energy is through solar cells by utilization of photoelectric effect. Formerly, silicon is considered as the efficient semiconducting materials in the solar cells owing to their higher power conversion efficiency (PCE), heat constancy and ease in access. In these days, utilization of silicon in silicon based solar cells has restricted because of certain factors like high cost, brittleness and fixed energy levels^4^. Recently bulk heterojunction (BHJ)^5^ OSCs have emerged as attractive candidates for global green energy sources due to their exceptional characteristics like flexibility, semitransparency, tunable energy levels, economic viability and potential commercial applications^6^. The OSCs have a blend of donor and acceptor molecules which link directly with each other through a backbone. OSCs bearing fullerene acceptors hold appealing assets including improved PCE, higher charge mobility^7^. In spite of these advantages, there have been found certain limitations associated with fullerene acceptors that limit their use^8^. To overcome such shortcomings of fullerene derivatives, non-fullerene acceptors (NFAs)^9^ materials with acceptor–donor–acceptor (A–D–A) backbone has given a great deal of attention^10^. The A–D–A diversity is of peculiar interest due to their unique properties like wide and efficient absorption bands and adjustable energy levels^11^. A–D–A combination comprises of central donor core unit which attached with two sideways electron deficient end capped acceptors through chemical bond. Narrowing the HOMO–LUMO band gap has proved the most effective strategy to enhance PCE and photovoltaic properties of non-fullerene based OSCs^12^. This can be brought successfully by choosing appropriate electron donor and withdrawing parts^13^.

Two imperative classes of non-fullerene acceptors have come under study recently such as Perylene diimide (PDI)^14,15^ and fused ring electron acceptors (FREAs)^16^. PCEs up to 9% have been reported in OSCs with PDI non fullerene acceptor. On the other hand, OSCs retaining non-covalently fused ring electron acceptors (NC-FREAs)^17^ configuration have fascinating properties because of their easy structural modulation^18,19^, strong optical absorption and enhanced PCE up to 14%^20^. Among FREAs, the most successful and widely discussed non fullerene acceptors type is ITIC^21^ with ladder type indacenodithienothiophene as a central core and with A–D–A type architecture enabling BHJ layer to enhance charge mobility. In comparison to traditional fullerene-based acceptor, ITIC types OSCs retain PCE up to 13%^22^. NFAs comparatively possess superior photovoltaic properties than their fullerene counterparts, that includes higher optical absorption, promising light capturing ability and tunable energy levels^23,24^. It is commonly observed that changing the molecular properties such as light absorbing capability, crystallinity, light absorption and energy levels by structural tailoring can effectively enhance the device performance^25,26^.

Considering the attractive qualities of fused ring non-fullerene acceptors based OSCs, seven new A–D–A type NC-FREAs (**MD2**–**MD8**) from parent **DOC2C6-2F** were designed. End capped acceptor modifications has been brought about in reference chromophore and their impact on the electronic and optical behavior of newly designed chromophores are studied. It is anticipated that these newly designed derivatives will play a vital part in the development of high efficacy OSCs cell materials.

## Results and discussion

**Figure 1:**
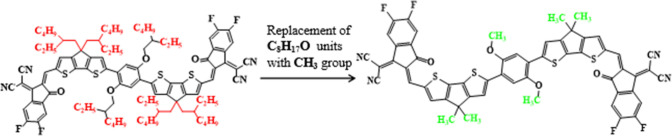
Modification of **DOC2C6**-**2F** into **MR1** via substitution of -CH_3_ group.

Aim behind the current study is to explore the photovoltaic response of NFAs type organic compounds. For this, **DOC2C6-2F**^27^ is selected to be used as a parent molecule comprised of central ladder-like 1,4-bis((2-ethylhexyl)peroxy)benzene core unit linked with donor (D) moiety along with two terminal electron capturing acceptor (A) components. In order to overcome the steric hinderance and computational cost caused by long alkyl chains in **DOC2C6-2F**, C_8_H_17_O substituted on the donor unit is replaced with methyl (–CH_3_) group as shown in Fig. 1. After this minute structural tailoring, the synthesized parent chromophore having A–D–A configuration is renamed as “**DOC2C6-2F**” to “**MR1**” reported as reference chromophore retaining the same A-D-A configuration.

**Figure 2:**
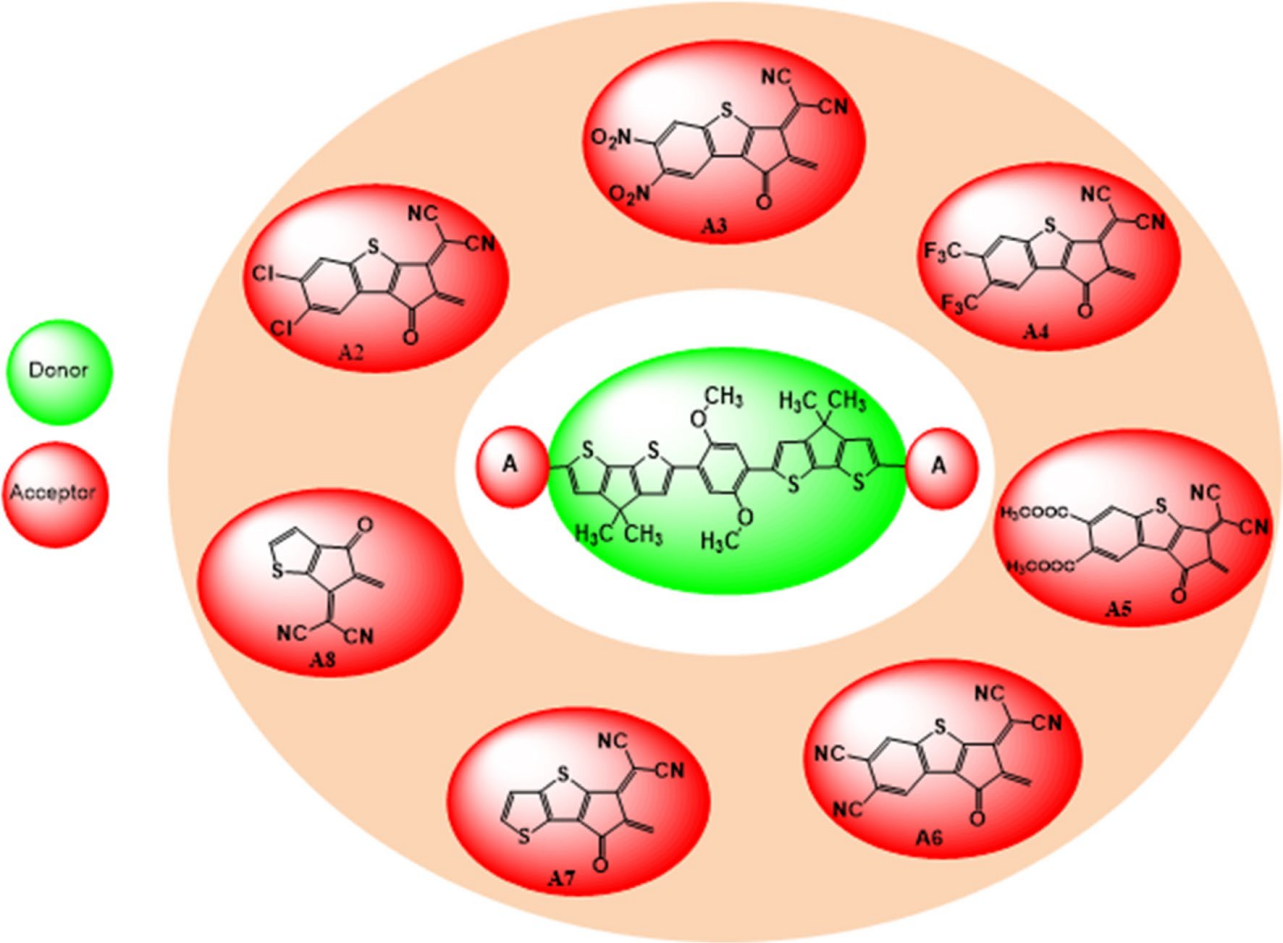
The sketch map of the reference and its derivative compounds.

**Figure 3:**
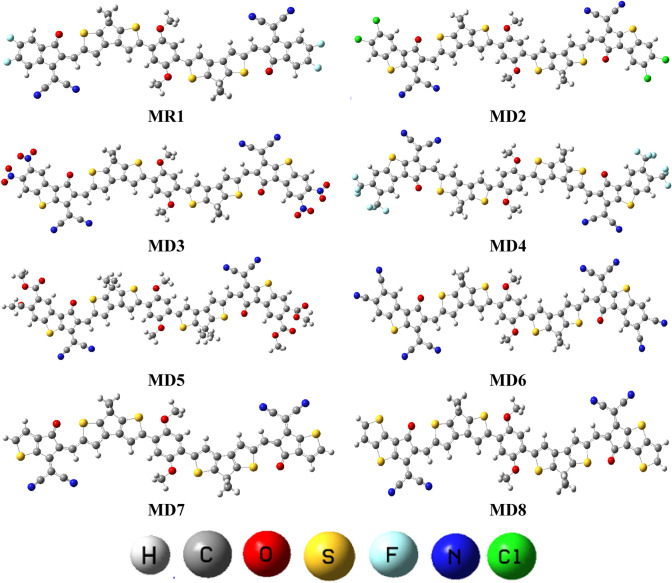
The optimized structures of all theoretically designed compounds. Figures are made with are made with the help of GaussView 5.0 and Gaussian 09 version D.01 (https://gaussian.com/g09citation/).

We changed the terminal acceptors of **DOC2C6-2F** with various well known end capped acceptors just to explore and boost the electronic properties of OSCs. **MD2**–**MD8** molecules having A–D–A configuration as shown in Fig. 2 and Fig. S1. The optimized structures of the **MR1** and its derivative **MD2**–**MD8** are depicted in Fig. 3.

### Frontier molecular orbital (FMO) analysis

FMO approach is believed to be an influential approach for determining the electronic characteristics of OSCs^28^. The HOMO and LUMO are regarded as valence and conduction bands according to the valance band theory. The energy difference between HOMO and LUMO has been elucidated as the band gap (*E*_g_)^29–32^. Efficiency reasonably depends upon the *E*_g_ as low energy band gap concludes large photovoltaic response of a compound and vice versa^33^. Computed energies and their *E*_g_ for designed chromophores are presented in Table 1.

**Table 1:** Energy of frontier molecular orbitals and *E*_LUMO_–*E*_HOMO_ of **MR1** and **MD2–MD8**.

Compounds	E_HOMO_	E_LUMO_	E_gap_
MR1	− 5.176	− 3.200	1.976
MD2	− 5.158	− 3.366	1.792
MD3	− 5.217	− 3.646	1.571
MD4	− 5.180	− 3.438	1.742
MD5	− 5.161	− 3.382	1.779
MD6	− 5.210	− 3.582	1.628
MD7	− 5.125	− 3.137	1.988
MD8	− 5.116	− 3.176	1.940

Units in eV.

**Figure 4:**
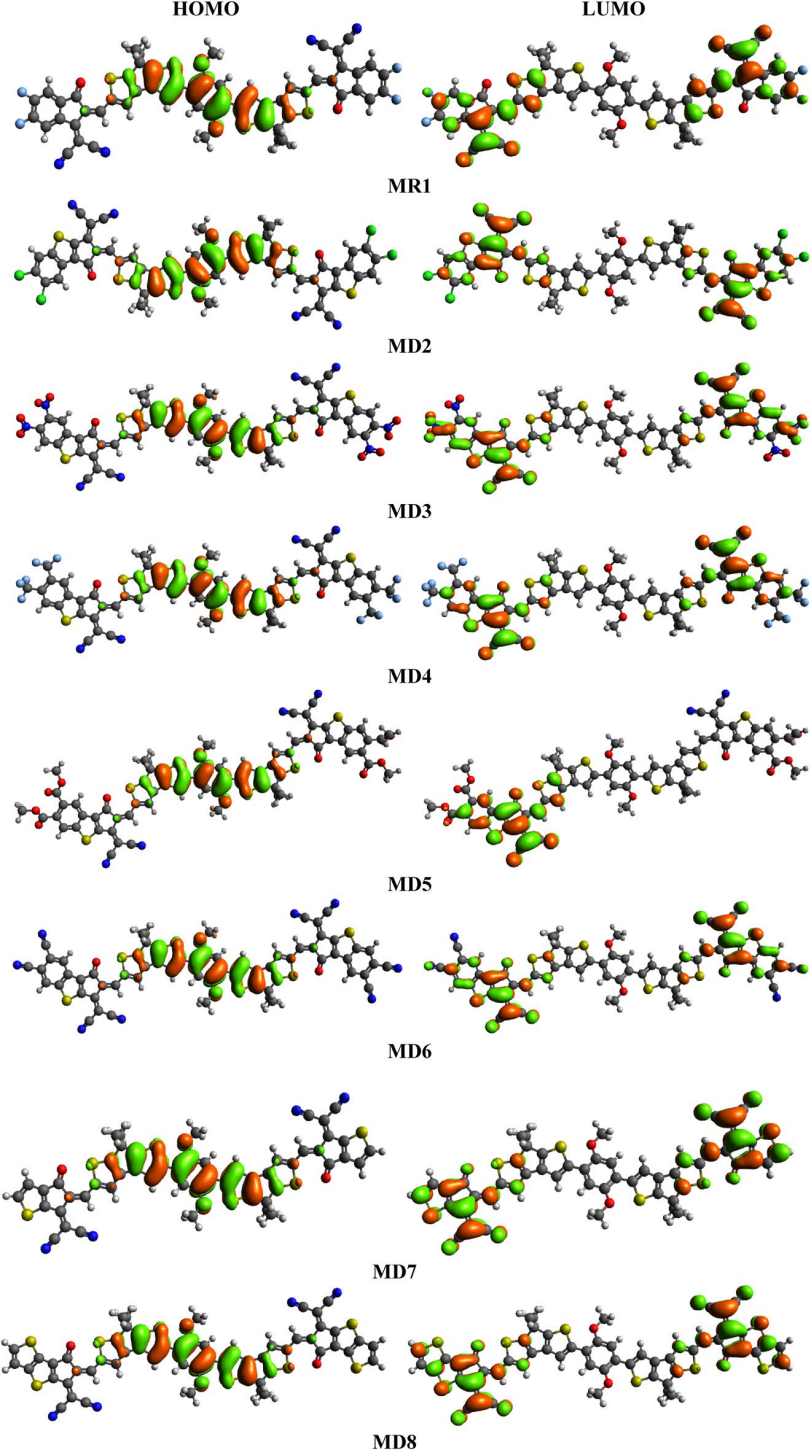
The FMOs (HOMOs & LUMOs) of the **MR1** and **MD2–MD8** drawn with the help of Avogadro software, Version 1.2.0. (http://avogadro.cc/). All out put files of entitled compounds were accomplished by Gaussian 09 version D.01 (https://gaussian.com/g09citation/).

In **MR1**, 1.976 eV* E*_g_ is noted having − 5.176 and − 3.200 eV HOMO and LUMO energies. Interestingly, a decrease in *E*_g_ has been noted for the designed chromophores (**MD2**–**MD6** and **MD8**) except **MD7** in which the *E*_g_ is larger than the reference compound (**MR1**). The energies for HOMO are noticed to be − 5.158, − 5.217, − 5.180, − 5.161, − 5.210, − 5.125 and − 5.116 eV for **MD2**-**MD8**, while for LUMO are − 3.200, − 3.366, − 3.646, − 3.438, − 3.382, − 3.582, − 3.137 and − 3.176 eV and the *E*_g_ between them is calculated as 1.792, 1.571, 1.742, 1.779, 1.628, 1.988 and 1.940 eV, respectively (Table 1). In **MD2**, a decline in *E*_g_ (1.792 eV) is found due to the introduction of thiophene ring and replacement of fluoro (–F) with chloro (–Cl) groups at the terminal acceptor moieties^34^. Another reason is the presence of thiophene ring as a result of which conjugation gets enhanced and *E*_g_ is observed to be reduced. The smallest *E*_g_ (1.571 eV) is noted for **MD3** among all the designed compounds in which the –Cl are altered with strong electron-withdrawing nitro (–NO_2_) groups at the acceptor parts. This decline in band gap is due to the greater –*I* effect of –NO_2_ as compared to the –Cl groups (NO_2_ > Cl). An increase in *E*_g_ (1.742 eV) is monitored in **MD4** in which the -NO_2_ is exchanged with the trifluoromethyl (–CF_3_) group. A slight enhancement in HOMO/LUMO band gap (1.779 eV) is expressed by **MD5** as compared to –CF_3_ because of the exchange of –CF_3_ with methyl acetate (–COOCH_3_) at the acceptor units. This increase in *E*_g_ is accredited to the lower electron withdrawing (− *I*) effect of –COOCH_3_ in comparison to the –CF_3_ group. The *E*_HOMO_–*E*_LUMO_ band gap (1.628 eV) is noticed to be reduced in **MD6**. It is because of the higher − *I* effect of –CN in comparison to –CF_3_ and –COOCH_3_. Also due to –CN, the charge transference rate is enhanced, resulting in lower *E*_g_ of orbitals. The highest *E*_g_ (1.988 eV) is found for **MD7** among all the designed molecules because of the removal of one fragment (pthalonitrile) of the acceptor moieties. Due to this reason the conjugation of the system is decreased as a result of which the band gap increased. *E*_g_ (1.940 eV) of **MD8** is retrieved to be less than the **MD7**, because of the incorporation of a thiophene ring at the peripheral acceptor units. As a result of which the conjugation in the molecule gets increased and the energy difference between the orbitals can be lowered. However, the overall descending trend of *E*_g_ is in the following order: **MD7 > MR1 > MD8 > MD2 > MD5 > MD4 > MD6 > MD3**. Furthermore, scheme of electronic cloud on the surface area^35^ of both **MR1** and **MD2**–**MD8** are represented in Fig. 4. Lowest *E*_*g*_ between the orbitals and efficient charge mobility from donor to end capped acceptors is inspected in **MD3** chromophore as compared to all other investigated chromophores which appeared to be an effective photovoltaic material.

### Density of states (DOS)

**Figure 5:**
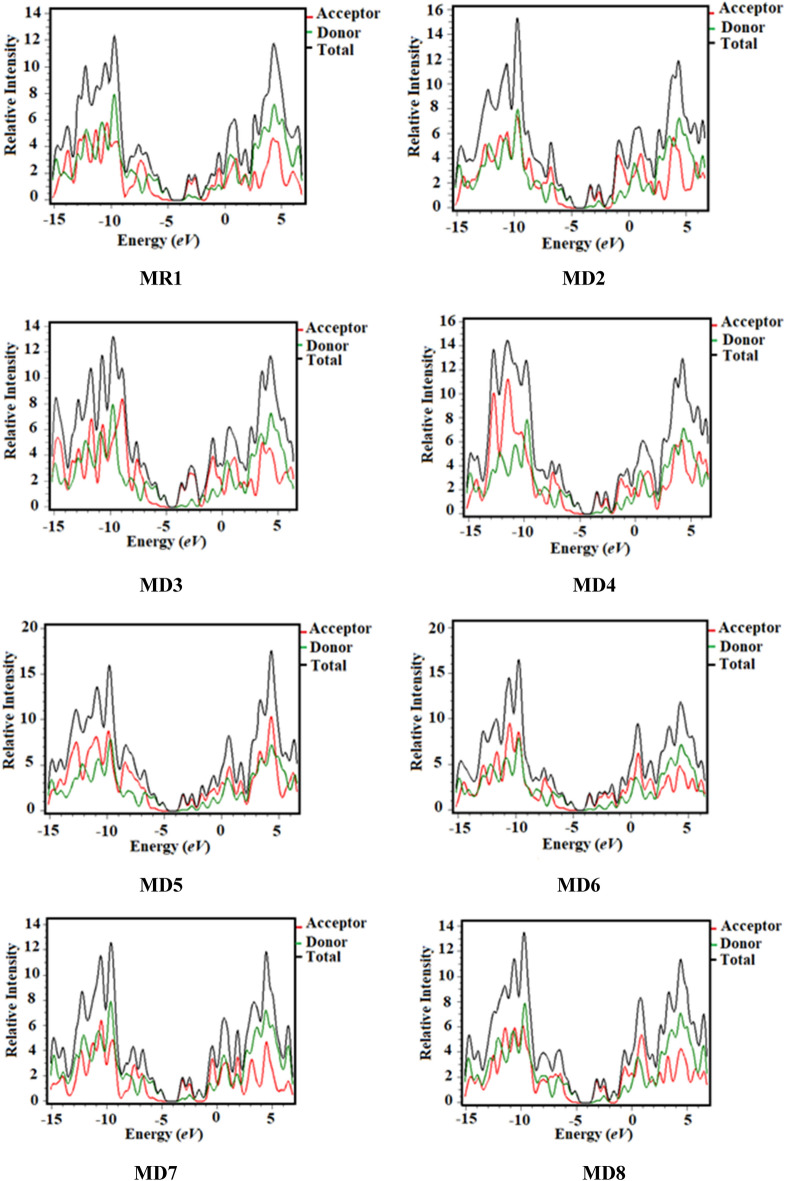
DOS spectra of the studied compounds (**MR1** and **MD2–MD8**) drawn by utilizing PyMOlyze 1.1 version (https://sourceforge.net/projects/pymolyze/). All out put files of entitled compounds were computed through Gaussian 09 version D.01 (https://gaussian.com/g09citation/).

The DOS approach has been performed to determine the effective contribution of each fragment to all over the molecular system having specific number of electronic states^36^. To predict the charge transfer path, we partitioned our compounds into two sections i.e., central donating core (D) and terminal acceptors (A). The DOS analysis of reference (**MR1**) and its derivatives was accomplished using B3LYP level of DFT with 6-31G(d,p) basis set to support FMO study (Fig. 4), DOS graphs are displayed in Fig. 5.

As depicted in the Fig. 4 as well as manifested from Fig. 5 that electronic cloud is distributed around HOMO and LUMO because of strong electron pulling character of terminal acceptor groups. Similarly, to LUMO donor contributes 97.1, 97.2, 97.1, 97.1, 97.2, 97.2 and 97% to HOMO while 8.2, 7.6, 8.4, 8.2, 8, 11.5 and 8.4% to LUMO in **MD2**–**MD8**, correspondingly. While acceptor contribution is 2.9, 2.8, 2.9, 2.9, 2.8, 2.8 and 3% to HOMO, while to LUMO 91.8, 92.4, 91.6, 91.8, 92, 88.5 and 91.6% for **MD2**–**MD8**, respectively. By these results, it is clear from DOS pictographs that the HOMO orbitals are positioned on the donating part shown with green colored peak which is found at − 6.5 eV*.* Similarly, LUMOs are majorly resided on the acceptor moiety in aforesaid chromophores with higher peak at 1.5 eV. Overall, the separation pattern of charge distribution reveals that huge amount of charge is shifted in **MD3** from donor towards acceptor moieties proving it to be most favorable candidate for non-fullerene OSCs applications.

### Optical properties

**Figure 6:**
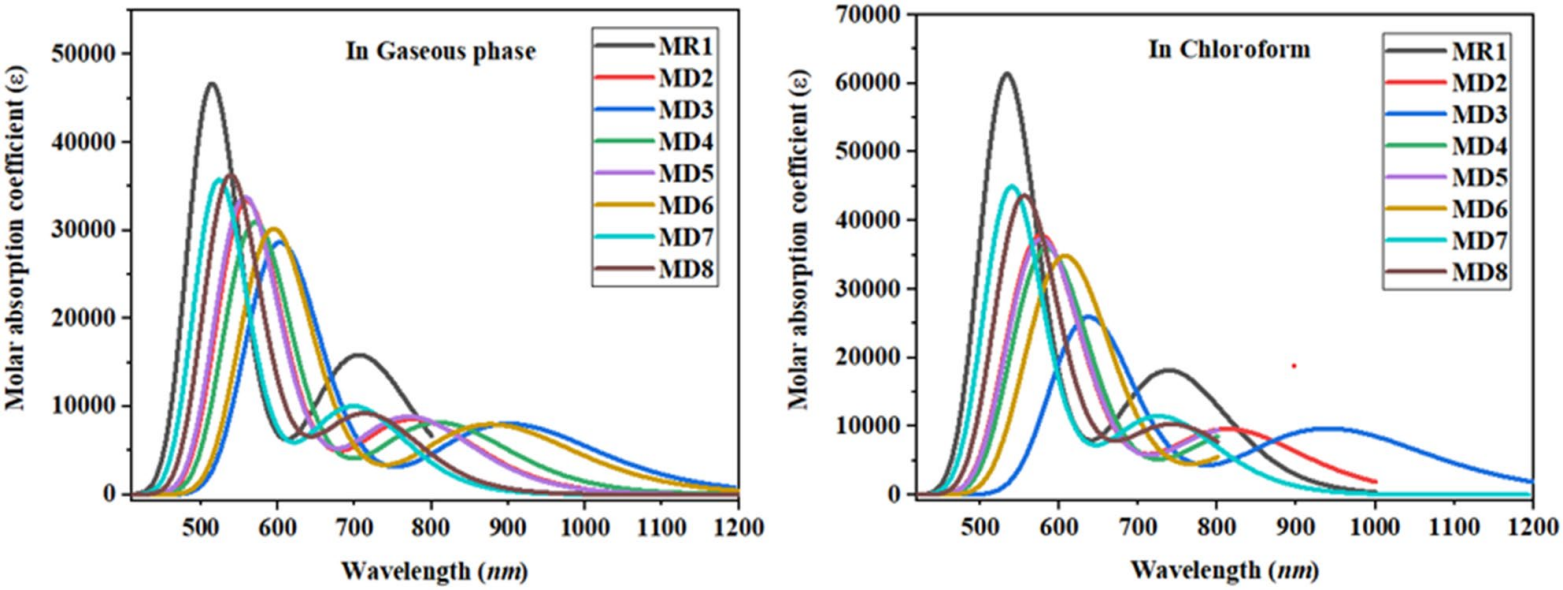
UV–visible absorption spectra of investigated molecules as their names can be seen in above graphs. These graphs are drawn by utilizing Origin Pro 8.5 version (https://originpro.informer.com/8.5/). All out put flies of entitled compounds were computed through Gaussian 09 version D.01 (https://gaussian.com/g09citation/).

To determine the effectiveness of OSCs, their optoelectronic capabilities are assessed by analyzing absorption spectra^37^. To characterize their photophysical properties, absorption spectra of entitled chromophores (**MR1** and **MD2–MD8**) were calculated in chloroform and gaseous phase at B3LYP/6-31G(d,p) level of theory. Numerous parameters like λ_max_, excitation energy, oscillator strengths (*f*) and molecular orbital contributions have been illustrated in Table 2 while UV–visible absorption spectrum of entitled molecules have been depicted in the Fig. 6. Generally strong electron accepting units leads to rise in the λ_max_ with lower excitation energies than the reference **MR1**^38^. This makes easy excitation between HOMO to LUMO causing higher charge transfer which ultimately lead to higher power conversion efficiency. The simulated λ_max_ of reference chromophore in chloroform solvent (738.221 nm) shows good harmony with the experimental recorded λ_max_ (743 nm)^27^ of reference **MR1** which also indicated the suitable selection of functional for DFT study.

**Table 2:** Calculated energies, wavelengths (λ_max_) and oscillation strengths for **MR1** and **MD2–MD8** in chloroform.

Compounds	λ (nm)	E (eV)	*f*_os_	Assignments
MR1	738.221	1.680	0.250	H → L (98%)
MD2	814.186	1.523	0.132	H → L (98%)
MD3	939.844	1.319	0.134	H → L (99%)
MD4	840.742	1.475	0.130	H → L (98%)
MD5	819.893	1.512	0.115	H → L (89%)
MD6	906.649	1.368	0.131	H → L (99%)
MD7	725.690	1.709	0.158	H → L (98%)
MD8	744.158	1.666	0.141	H → L (97%)

The λ_max_ ranges from 939.844 to 725.690 nm for entitled chromophores in chloroform. Among all designed molecules **MD3** shows the maximum absorption wavelength of 939.844 nm that is attributed to highly electron accepting end capped Nitro group. Lower E_g_ and lower transition energies, both of these factors contribute to greater charge mobility and high-power conversion efficiency^38^. Consequently, the lower value of λ_max_ has been observed in the case of **MD7** (725.690 nm) with excitation energy of 1.709 eV*.* All the explored chromophores possess higher absorption range than reference **MR1** (738.221 nm) except **MD7**. It clear from Table 2 that **MD2**, **MD4**, **MD5**, **MD6**, and **MD8** have maximum absorption values of 814.186, 840.742, 819.893, 906.649 and 744.158 nm respectively. Results indicate that λmax of all studied compounds lie in the visible region. Classification of entitled chromophores with respect to decreasing λ_max_ in chloroform is **MD3 > MD6 > MD4 > MD5 > MD2 > MD8 > MR1 > MD7**.

In gaseous phase, all the investigated chromophores almost maintained the same order and properties as in chloroform. In gas phase, calculated λ_max_ of all enlisted chromophores lies in the range of 900.067–698.109 nm. A small decrease in the values of λ_max_ of entitled molecules in gas phase is noted which might be due to the solvent effect as shown in Table 3. In gas phase maximum absorbed wavelength decreases in the following order **MD3 > MD6 > MD4 > MD2 > MD5 > MD8 > MR1 > MD7**. The above trend concludes that **MD3** being the red shifted of all in absorption spectrum of both chloroform and gaseous phase would be an efficient OSC material.

**Table 3:** Computed energy, wavelength (λ_max_) and oscillator strength for **MR1** and **MD2–MD8** in gaseous phase.

Compounds	λ_max_ (nm)	E (eV)	*f*_os_	MO contributions
MR1	705.337	1.758	0.219	H → L (99%)
MD2	776.162	1.597	0.118	H → L (98%)
MD3	900.067	1.378	0.111	H → L (99%)
MD4	808.927	1.533	0.113	H → L (98%)
MD5	775.531	1.599	0.081	H → L (96%)
MD6	877.081	1.414	0.110	H → L (99%)
MD7	698.109	1.776	0.138	H → L (98%)
MD8	713.742	1.737	0.127	H → L (98%)

HOMO = H, LUMO = L, ***f* = **oscillator strength, MO = molecular orbital.

Another crucial factor that can influence the electron mobility of entitled chromophores is excitation energy^39^. Works concludes that molecule with small excitation energy hold improved charged mobilities, easy transition from valence to conduction band and higher PCE. The excitation energy values of all designed molecules are found less than the reference compound **MR1** except **MD7**. The lowest computed excitation energy of **MD3** is attributed to the strong electron pulling character of nitro group.

The preceding discussion concludes that all the designed molecules specifically **MD3** contain lower excitation energy and higher absorption thus having excellent potential to use in non-fullerene OSCs.

### Reorganization energy

Reorganization energy ($\lambda$) is an imperative quantity in determining charge transfer characteristics among molecular structures while designing efficient materials for OSCs. The potential OSCs is mainly dependent upon the reorganization energy, which is actually the electron and hole transport ability of different materials. Generally, materials having good charge transport ability exhibit substantial optoelectronic properties^40^. Reorganization energy and charge transfer capability have inverse relationship to each other, such that lower $\lambda$ causes higher charge mobilities^41^. So, materials having higher charge mobilities ultimately have lower $\lambda$ with widespread potential usage in non-fullerene OSCs. Reorganization energy varies with certain factors like nature of the molecule and their configuration but to a greater extent it is affected by geometries of cations and anions. Electron (*λ*_*e*_) mobility has an association with anionic geometry while hole in acceptor material is represented by cationic geometry. Mainly, $\lambda$ represents the charge transfer from donor to acceptor unit^42^. Overall, $\lambda$ is categorized into two divisions; internal reorganization energy (λ_int_) and external reorganization energy (λ_ext_). The first one i.e. λ_int_ has founded its concerns with internal structural changes while λ_ext_ deals with the polarization influence. In this study, factor of external reorganization has not taken into consideration as external environment does not contribute much, so only λ_int_ is focused^43^.

In this work, we theoretically calculated the reorganization energies of **MR1** and **MD2**–**MD8** utilizing Eqs. (1) and (2) and results are tabulated in Table 4.

**Table 4:** Computed reorganization energies of **MR1** and **MD2**–**MD8** chromophores.

Compounds	*λ*_*e*_	*λ*_*h*_
MR1	− 0.001702	− 0.004788
MD2	0.000152	− 0.000132
MD3	0.000054	− 0.000266
MD4	0.000133	− 0.000264
MD5	0.000129	− 0.000187
MD6	0.000123	− 0.224763
MD7	0.000147	0.000025
MD8	0.000182	0.000029

From Table 4, it is clear that *λ*_*e*_ of reference compound **MR1** is − 0.001702 eV. The computed electron mobilities of all entitled chromophores (**MD2–MD8**) are 0.000152, 0.000054, 0.000133 and 0.000129, 0.000123, 0.00014.7 and 0.000182 eV correspondingly. Reference compound **MR1** exhibit lower reorganization energy of electron (*λ*_*e*_) indicating the higher electron transport abilities between donor and acceptor part. Among all the derivatives, **MD3** has the least value of *λ*_*e*_ which denotes the higher electron transport rate between HOMO and LUMO. Similarly, **MD6** and **MD5** considerably have better electron mobilities owing to their smaller value of *λ*_*e*_. The *λ*_*e*_ values of all entitled chromophores decreases in the following order **MD8 > MD2 > MD7 > MD4 > MD5 > MD6 > MD3 > MR1**.

Similarly, the theoretical calculated *λ*_*h*_ of **MR1** is − 0.004788 eV. The **MD2–MD8** show *λ*_*h*_ value of − 0.000132, − 0.000266, − 0.000264 and − 0.000187, − 0.224763, 0.000025 and 0.000029 eV correspondingly. Among all, **MD3** is the finest molecule for hole transport capability owing to its smallest value of *λ*_*h*_ − 0.000266 eV. The descending order of *λ*_*h*_ of all entitled chromophores is **MD6 > MR1 > MD7 > MD4 > MD5 > MD2 > MD8 > MD3**.

The overall discussion concludes that, **MD3** is the molecule with best electron and hole transport abilities thus appeared to be the fine candidate to utilize in non-fullerene organic solar cell future applications.

### Open circuit voltage (*V*_*oc*_)

**Figure 7:**
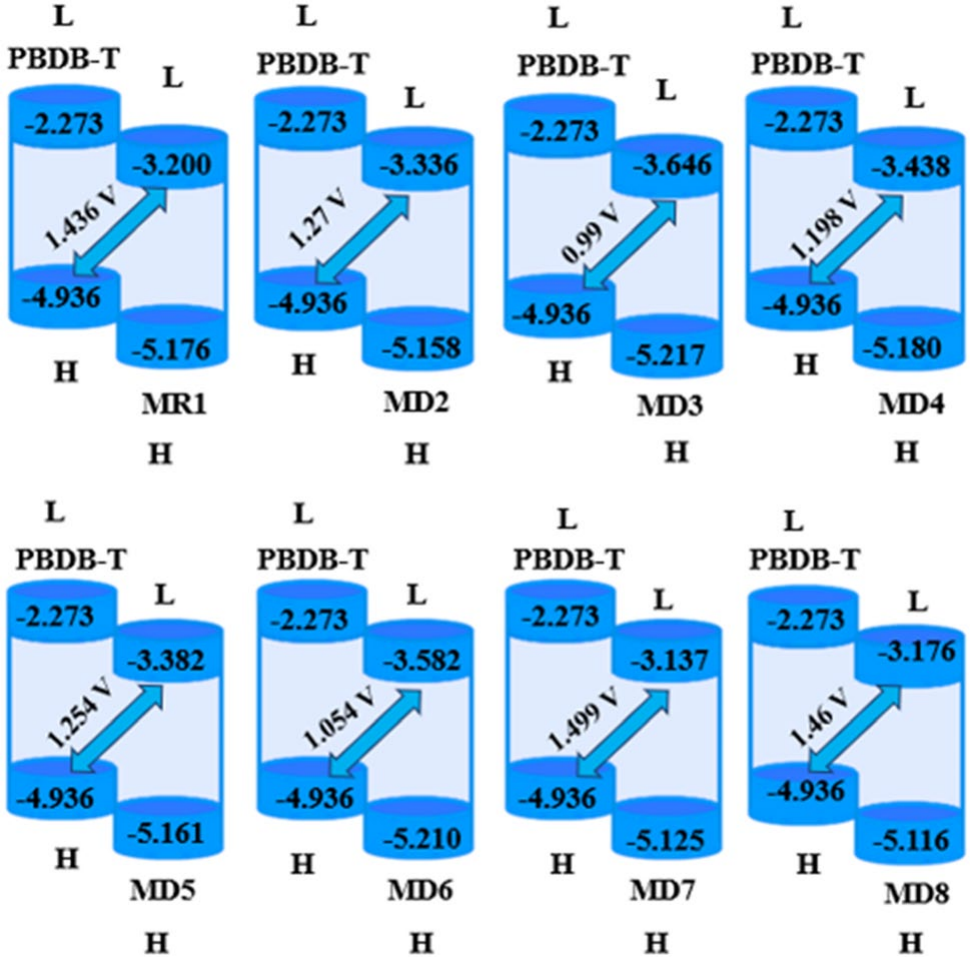
Diagrammatic illustration of *V*_*oc*_ for investigated molecules drawn with the aid of power point. All out put files of entitled compounds were accomplished by Gaussian 09 version D.01 (https://gaussian.com/g09citation/).

Open circuit voltage being a vital parameter well indicates the performance and working mechanics of the semiconductor materials like OSCs^44^. It represents the total magnitude of current that can be taken away from any optically active device at null voltage^45^. Certain influential factors affecting the *Voc* are; light source, light intensity, external fluorescence proficiency, recombination of charge carriers and various environmental features^46^. To attain better *Voc*, LUMO value of acceptor should be greater as compared to HOMO of donor moiety as it lowers band gap^47^. Here in this manuscript, we related the results of LUMO of our tailored molecules with the HOMO of renowned polymer **PBDB-T**^48^ having − 4.936 eV as E_HOMO_ of donor polymer and results are summarized in Fig. 7. The computed *Voc* results of **MR1** and **MD2**–**MD8** by means of Scharber equation^49^ are tabulated in Table 5.

1 $$Voc=(\left|{\text{E}}_{\text{HOMO}}^{\text{D}}\right|-\left|{\text{E}}_{\text{LUMO}}^{\text{A}}\right|)-0.3$$

Here, E is energy level, e indicates charge present on each molecule, and 0.3 is the empirical constant.

**Table 5:** ***V***_***OC***_ of entitled molecules.

Compounds	$\Delta$E (eV)	*V*_*OC*_ (V)
MR1	1.736	1.436
MD2	1.57	1.27
MD3	1.29	0.99
MD4	1.498	1.198
MD5	1.554	1.254
MD6	1.354	1.054
MD7	1.799	1.499
MD8	1.76	1.46

$$\Delta \mathbf{E}={\text{E}}_{\text{LUMO}}^{\text{A}}-{\text{E}}_{\text{HOMO}}^{\text{D}}.$$

The *Voc* value for **MR1** and **MD2**–**MD8** is 1.436, 1.27, 0.99, 1.198, 1.254, 1.054, 1.499 and 1.46 V respectively. A good harmony is seen between the simulated (0.85 V)^27^ and experimental value (1.436 V) of reference chromophore which supports that suitable selection of functional for computational analysis. The computed *Voc* value of compound **MD3** is 0.99 V. The *Voc* of **MR1** with regards to HOMO_donor_–LUMO_MR1_ is 1.436 V. **MD7** showed the highest *Voc* (1.499 V). Except **MD7** and **MD8**, all the designed molecules showed lower values of *Voc* than reference chromophore **MR1**. The decreasing order of *Voc* of designed chromophores with respect to HOMO_donor_–LUMO_acceptor_ is **MD7** > **MD8** > **MR1** > **MD2** > **MD5** > **MD4** > **MD6** > **MD3**. The band gap is an intrinsic property of semiconductors and eventually has a direct influence on the photovoltaic response of a compound. Band gap energy is the energy required for excitation from the highest occupied molecular orbital (HOMO) towards the lowest unoccupied molecular orbital (LUMO). Having low band gap means easy excitation or less energy will be required to excite electrons from HOMO to LUMO. If band gap is low, most photons will have more energy than necessary to excite electrons across the band gap generating more electricity with higher efficiency and large photovoltaic response. Herein, we reported our all-designed molecules which are of acceptor nature and are used in making bulk heterojunction devices by blended with polymer (PBDB-T) of donor type. When a complex of donor–acceptor formed we found that our all-designed chromophores have low energy of LUMO in comparison to LUMO of donor polymer. So, there will be a better transition from HOMO of donor polymer to the low lying LUMO of our designed molecules in comparison to the LUMO of donor polymer with more knocking out of electrons and thus enhanced efficiencies. The orbital energy diagram with respect to **PBDB-T** of all molecules is shown in Fig. 6.

### Transition density matrix (TDM) analysis

**Figure 8:**
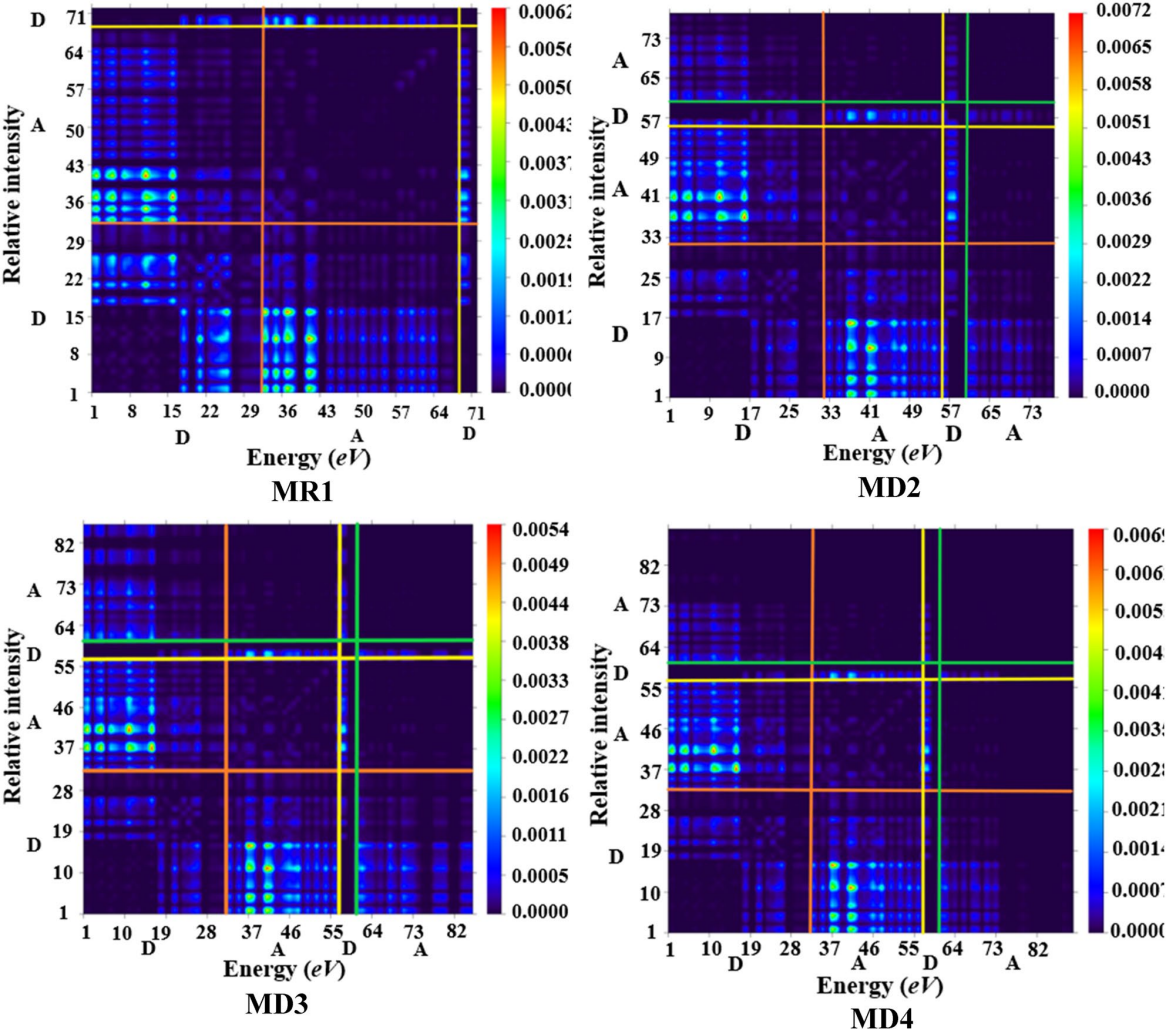

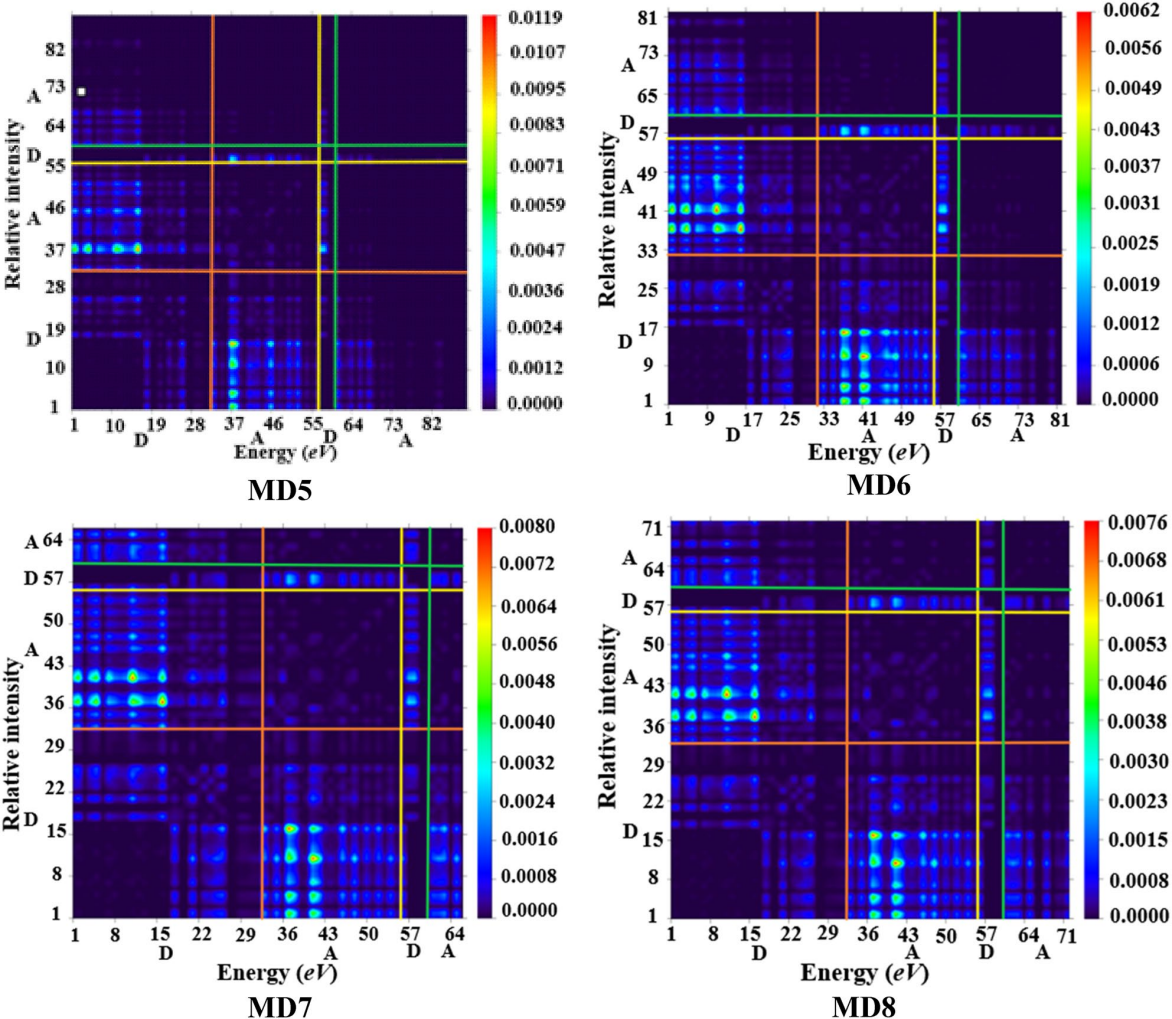
TDM for the entitled compounds (**MR1**and **MD2–MD8**). These were drawn with the help of Multiwfn 3.7sofware (http://sobereva.com/multiwfn/). All out put files of designed compounds were accomplished by Gaussian 09 version D.01 (https://gaussian.com/g09citation/).

The interpretation of transition processes^50^ and extent of intramolecular charge transfer within a conjugated system can be efficiently determined using transition density matrix (TDM). This analysis gives a pictorial display of interactions between donor and acceptor entities in excited state^51^ with three dimensional plots having sufficient color differentiation i.e., blue region displays^52^. In addition to this, the electron–hole localization and the extent of electronic movements in the specific regions of entitled chromophores (**MR1** and **MD2–MD8**) are indicated^41^. The behavior of transitions of entitled chromophores was investigated with B3LYP/6-31G(d,p) level of theory. In all the investigated compounds, the $\uppi$-conjugation is absent and instead an A–D–A configuration is retained (Fig. 8). The H-atoms are neglected due to their minute influence in transitions as compared to the other atoms. The Fig. 8 represents the results obtained for TDM analysis for all the studied compounds.

To simplify the calculations in our present report, we categorized all structures (**MR1** and **MD2–MD8**) into two different areas i.e., donor (D) and acceptor (A). From TDM diagrams, it is evident that electron coherence is largely present on end capped acceptor portions as compared to donor entity. According to the results obtained from TDM heat maps, it has been observed that the electronic charge is efficiently transferred in diagonal way from the donor towards acceptor in all derivatives without trapping, showing a charge coherence. This makes clear that, donor and end-capped acceptor units efficiently donate and withdraw the electron density respectively. The most prominent charge shift is observed in compound **MD3**. This might be due to the presence of strong electron withdrawing nitro (–NO_2_) group at acceptor units. Therefore, we can infer from the efficient charge transfer response that **MD**3 chromophore could be conveniently utilized for the development of solar cell devices in the future^53^.

### Exciton binding energy (*E*_b_) analysis

Another special feature related to TDMs is binding energy (*E*_*b*_) that is used to assess the photovoltaic response of OSCs. It is an important parameter for the estimation of exciton dissociation capacity and columbic force interaction, as *E*_*b*_ declines, the columbic forces between hole and electron also decreases^54^. Both these factors led to higher exciton dissociation in excited state^55,56^. The *E*_*b*_ of investigated molecules **MR1** and **MD2**–**MD8** is calculated from the difference of *E*_gap_ (LUMO–HOMO) and energy of optimization (*E*_opt_) ^43^as represented in the Eq. (2).

2 $$E_{b} = E_{L - H} - E_{opt}$$

In Eq. (2), *E*_*b*_ is binding energy, E_L–H_ is the band gap between LUMO and HOMO and E_opt_ represents the first singlet to singlet excitation state energy^57^. Calculated outcomes are tabulated in Table 6.

**Table 6:** Calculated first singlet to singlet excitation state energy (*E*_*opt*_) and binding energy (*E*_*b*_) of inspected compounds.

Compounds	*E*_*L–H*_	*E*_*opt*_	*E*_*b*_
MR1	1.976	1.680	0.296
MD2	1.792	1.523	0.269
MD3	1.571	1.319	0.252
MD4	1.742	1.475	0.267
MD5	1.779	1.512	0.267
MD6	1.628	1.368	0.260
MD7	1.988	1.709	0.279
MD8	1.940	1.666	0.274

Units in eV.

According to Table 6, **MD3** has given out lowest value of binding energy (0.252 eV*)* giving highest number of charges. In comparison to **MR1**, other molecules have smaller *E*_b_ providing reasonable justification for further calculations. Generally, structures with 1.9 eV or lower E_b_ are taken as efficient OSC materials with impressive *V*_*oc*_. Interestingly, our all-investigated molecules possess *E*_*b*_ values smaller than 1.9 eV*.* The descending sequence of investigated chromophores related to *E*_*b*_ is as **MR1 > MD7 > MD8 > MD2 > MD4 > MD5 > MD6 > MD3**. So, it is clear that among all investigated chromophores, **MD3** is the molecule with lowest value of *E*_*b*_ justifying greater magnitude of dissociation into free electrons with superior photo-electronic properties that describe it to be effective material for OSCs.

## Conclusion

In this study, sophisticated quantum chemical procedures have utilized to examine photo-voltaic, photophysical and electronic capabilities of the explored derivatives. By performing structural modification in reference molecule (**MR1**) by end-capped acceptor moieties, seven new compounds (**MD2**–**MD8**) are designed. This terminal structural tailoring has proved to be the most important tactic to gain impressive photovoltaic compounds with improved opto-electronic possessions for efficient OSCs. Decrease in the energy gap of designed molecules (1.571–1.988 eV) is observed as compared with reference (1.976 eV) with efficient electron transfer rate from HOMO to LUMO, which is further, supported by DOS data. Further, the designed compounds exhibited a red shift in the visible region (939.844–725.690 nm) in chloroform solvent in comparison to reference **MR1** (*λ*_max_ = 738.221 nm). Moreover, the *V*_*oc*_ is also estimated with regarding to ${\text{HOMO}}_{\mathbf{P}\mathbf{B}\mathbf{D}\mathbf{B}-\mathbf{T}}-{\text{LUMO}}_{\text{Acceptor}}$. Binding energy (*E*_b_) of designed systems is smaller than **MR1** molecule, that elucidated the greater exciton dissociation rate, results in high-power conversion efficiency of fullerene free acceptor groups in OSCs.

## Computational procedure

The entire theoretical calculations of the current work were computed employing the Gaussian 09^58^ package. In order to visualize the designing of non-fullerene acceptor type chromophores and for the featuring of DFT based calculations, Gauss View 5.0^59^ was utilized. TD-DFT calculations were carried out for the optimization of reference chromophore (**MR1**) by adapting different level of DFT such as B3LYP^60^, CAM-B3LYP^61^, MPW1PW91^62^, M06^63^, M06.2X^64^ with 6-31G(d,p) basis set. Computed maximum absorbed wavelength of reference chromophore **MR1** at aforementioned functionals were compared with the experimental reported results for selecting valid theoretical method. λ_max_ values of reference compound obtained using these functionals are 738.211, 439.380, 579.338, 565.028 and 446.436 nm correspondingly while experimental recorded λ_max_ of parent chromophore, **DOC2C6-2F** is 743 nm^27^. Functional B3LYP with 6-31G(d,p) had been shown the best agreement of DFT with experimental UV–visible results of **MR1** as displayed in Fig. 8 and was selected for computational analysis ahead.

Moreover, to visualize the effectiveness of enlisted chromophores, the electronic computations of the TDM, UV–Vis, E_b_, *V*_*oc*_, *E*_HOMO_-*E*_LUMO_ band gap analysis and reorganization energies are calculated employing the same B3LYP/6-31G(d,p) level of theory.

Overall, $\lambda$ is categorized into two main divisions. The first one i.e. internal reorganization has founded its concerns with internal structural variations while λ_ext_ deals with the polarization influence in the external environment. In this study, factor of external reorganization has not taken into consideration as external environment does not contribute much, so only λ_int_ is focused^43^.

Hence, for the calculations of reorganization of electron (*λ*_*e*_) and hole (*λ*_*h*_) following Eqs. (3) and (4) are utilized.

3 $$\lambda_{e} = [E_{0}^{ - } - E_{ - } ] + [E_{ - }^{0} - E_{0} ]$$

4 $$\lambda_{h} = [E_{0}^{ + } - E_{ + } ] + [E_{ + }^{0} - E_{0} ]$$

**Figure 9:**
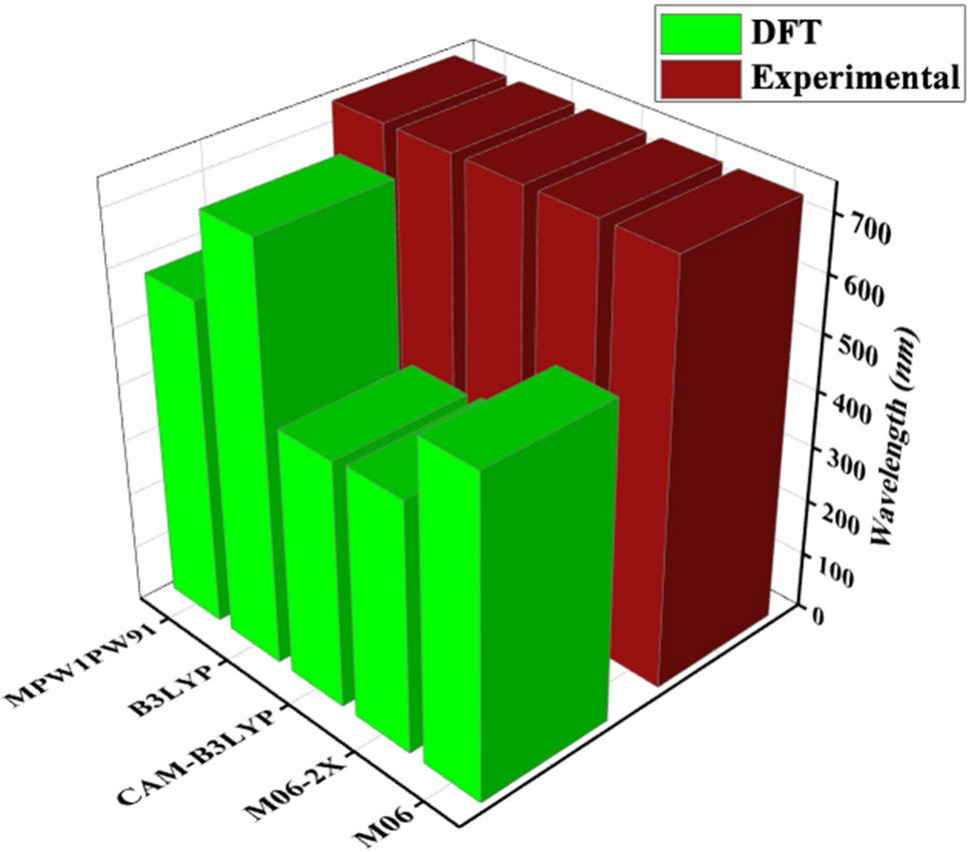
Comparison between DFT and experimental UV–visible results of **MR1** at various levels in chloroform solvent. These graphs are drawn by utilizing Origin Pro 8.5 version (https://originpro.informer.com/8.5/). All out put flies of entitled compounds were computed through Gaussian 09 version D.01 (https://gaussian.com/g09citation/).

While ${E}_{0}^{-}$ and ${E}_{0}^{+}$ are the energies of neutral molecule via anion and cation optimized structures correspondingly, ${E}_{-}$ and ${E}_{+}$ represents anionic and cationic energy, ${E}_{-}^{0}$ and ${E}_{+}^{0}$ are the energies of cationic and anionic structures via structures of neutral molecule (Fig. 9).

## Supplementary Information

Supplementary Information.

## Data Availability

All data generated or analyzed during this study are included in this published article and its supplementary information files.
