# Supplementary material for: First theoretical framework for highly efficient photovoltaic parameters by structural modification with benzothiophene-incorporated acceptors in dithiophene based chromophores
Source: Sci Rep. 2022 Nov 23;12:20148. doi: 10.1038/s41598-022-24087-8 (PMC9684146; doi:10.1038/s41598-022-24087-8)
Supplement: Supplementary file 1 — Supplementary Information. [file 41598_2022_24087_MOESM1_ESM.docx]

**Supplementary information**

**First theoretical framework for highly efficient photovoltaic parameters by structural modification with benzothiophene-incorporated acceptors in dithiophene based chromophores**

Muhammad Khalid,^1,2^ Rameez Ahmed,^1,2^ Iqra shafiq,^1,2^ , Muhammad Arshad,^3^ Muhammad Adnan Asghar,^4^ Khurram Shahzad Munawar,^5^ Muhammad Imran^6^, Ataualpa A. C. Braga,^7^

^1^Institute of Chemistry, Khwaja Fareed University of Engineering & Information Technology, Rahim Yar Khan, 64200, Pakistan

^2^Centre for Theoretical and Computational Research, Khwaja Fareed University of Engineering & Information Technology, Rahim Yar Khan, 64200, Pakistan

^3^Department of Chemical Engineering, College of Engineering, King Khalid University, Abha, Saudi Arabia

^4^Department of Chemistry, Division of Science and Technology, University of Education Lahore, Pakistan

^5^Department of Chemistry, University of Mianwali, Mianwali 42200, Pakistan

^6^Department of Chemistry, Faculty of Science, King Khalid University, P.O. Box 9004, Abha 61413, Saudi Arabia.

^7^Departamento de Química Fundamental, Instituto de Química, Universidade de São Paulo, Av. Prof. Lineu Prestes, 748, São Paulo, 05508-000, Brazil

*Corresponding authors E-mail addresses:

Dr. Muhammad Khalid (muhammad.khalid@kfueit.edu.pk; [khalid@iq.usp.br](mailto:khalid@iq.usp.br))

**Table S1**: Cartesian coordinates of **MR1** chromophore.

| **Atom** | **X-axis** | **Y-axis** | **Z-axis** |
| --- | --- | --- | --- |
| C | -0.186506 | 1.33797 | 0.36321 |
| C | -1.316556 | 0.566195 | 0.006339 |
| C | -1.084856 | -0.771509 | -0.36234 |
| C | 0.186501 | -1.337914 | -0.362378 |
| C | 1.316547 | -0.566127 | -0.005509 |
| C | 1.084855 | 0.771562 | 0.363191 |
| H | -1.928588 | -1.36685 | -0.682331 |
| H | 1.92859 | 1.366871 | 0.683235 |
| C | 2.691088 | -1.075291 | -0.027719 |
| C | 3.84675 | -0.314595 | -0.057432 |
| S | 3.088341 | -2.802454 | 0.067558 |
| C | 5.024381 | -1.099971 | 0.017615 |
| H | 3.839206 | 0.763991 | -0.15275 |
| C | 4.774802 | -2.453752 | 0.100682 |
| C | -2.691104 | 1.075355 | 0.028519 |
| C | -3.846775 | 0.314646 | 0.057772 |
| S | -3.088375 | 2.802561 | -0.066109 |
| C | -5.024404 | 1.100038 | -0.017073 |
| H | -3.839254 | -0.76398 | 0.152626 |
| C | -4.774827 | 2.453854 | -0.099542 |
| C | -8.755485 | 0.373049 | 0.000501 |
| C | -7.43344 | -0.10226 | 0.03861 |
| C | -6.478023 | 0.919496 | -0.012838 |
| H | -7.21764 | -1.163182 | 0.100951 |
| C | 6.477999 | -0.919443 | 0.013134 |
| C | 7.060888 | -2.182959 | 0.09017 |
| C | 8.75546 | -0.373013 | -0.000627 |
| S | -8.774298 | 2.149874 | -0.101954 |
| S | 8.774276 | -2.149797 | 0.102524 |
| H | 7.217616 | 1.163182 | -0.101641 |
| C | 7.433415 | 0.102287 | -0.038856 |
| C | -7.060911 | 2.183042 | -0.089393 |
| C | -6.035727 | 3.311124 | -0.146879 |
| C | 6.035702 | -3.311013 | 0.14823 |
| C | -6.146806 | 4.119241 | -1.459824 |
| H | -7.102561 | 4.651024 | -1.505093 |
| H | -5.342512 | 4.859519 | -1.514977 |
| H | -6.071907 | 3.464192 | -2.331225 |
| C | -6.151375 | 4.249647 | 1.076159 |
| H | -5.346184 | 4.990825 | 1.05867 |
| H | -7.106477 | 4.784201 | 1.061957 |
| H | -6.081272 | 3.687233 | 2.010406 |
| C | 6.151216 | -4.250027 | -1.074444 |
| H | 7.106315 | -4.784586 | -1.06012 |
| H | 5.34602 | -4.991191 | -1.056579 |
| H | 6.081028 | -3.687986 | -2.008908 |
| C | 6.146922 | -4.118598 | 1.461487 |
| H | 5.342639 | -4.858859 | 1.517026 |
| H | 7.102686 | -4.650358 | 1.506871 |
| H | 6.072113 | -3.463197 | 2.332632 |
| C | -9.846383 | -0.535026 | 0.041369 |
| C | -11.217852 | -0.397786 | 0.022495 |
| H | -9.477853 | -1.553054 | 0.100483 |
| C | 9.846362 | 0.535033 | -0.041995 |
| C | 11.217828 | 0.397769 | -0.023161 |
| H | 9.477841 | 1.553036 | -0.101562 |
| C | -12.205066 | -1.483196 | 0.073022 |
| C | -11.981748 | -2.846987 | 0.136127 |
| C | -11.957023 | 0.882126 | -0.047868 |
| C | 11.956953 | -0.882131 | 0.047904 |
| C | 12.205082 | 1.483117 | -0.074324 |
| C | 11.981839 | 2.846881 | -0.138287 |
| O | -11.493643 | 2.015185 | -0.107041 |
| O | 11.493527 | -2.015139 | 0.107694 |
| C | -15.744253 | 0.867767 | -0.051491 |
| C | -15.917011 | -0.522394 | 0.021615 |
| C | -14.840868 | -1.399337 | 0.068119 |
| C | -13.55091 | -0.853343 | 0.040105 |
| C | -13.39743 | 0.541957 | -0.032618 |
| C | -14.472852 | 1.420036 | -0.079535 |
| H | -15.04676 | -2.459336 | 0.123821 |
| H | -14.327114 | 2.493022 | -0.135467 |
| C | 14.840877 | 1.399167 | -0.069316 |
| C | 13.550901 | 0.853232 | -0.041017 |
| C | 13.397372 | -0.542021 | 0.032489 |
| C | 14.472765 | -1.420109 | 0.07992 |
| C | 15.744185 | -0.867898 | 0.051591 |
| C | 15.91699 | 0.522215 | -0.022295 |
| H | 15.046806 | 2.459129 | -0.125609 |
| H | 14.32699 | -2.493058 | 0.136451 |
| C | -13.035112 | -3.811759 | 0.18068 |
| N | -13.867474 | -4.626047 | 0.218171 |
| C | -10.685574 | -3.448515 | 0.161359 |
| N | -9.645165 | -3.973305 | 0.182694 |
| C | 10.685707 | 3.448479 | -0.16396 |
| N | 9.64535 | 3.973358 | -0.185665 |
| C | 13.035268 | 3.811558 | -0.183387 |
| N | 13.867693 | 4.62576 | -0.221337 |
| F | -17.166652 | -1.000904 | 0.046156 |
| F | -16.832775 | 1.644587 | -0.093165 |
| F | 16.83268 | -1.644731 | 0.093728 |
| F | 17.166647 | 1.000672 | -0.047076 |
| O | -0.429185 | 2.636633 | 0.712437 |
| O | 0.429208 | -2.636575 | -0.711526 |
| C | 0.660587 | 3.479955 | 1.053449 |
| H | 1.180839 | 3.12156 | 1.950222 |
| H | 1.37735 | 3.565272 | 0.22741 |
| H | 0.226772 | 4.459291 | 1.257729 |
| C | -0.660452 | -3.4798 | -1.053106 |
| H | -1.377442 | -3.565412 | -0.227292 |
| H | -1.180467 | -3.121127 | -1.949905 |
| H | -0.226556 | -4.45906 | -1.257575 |

**Table S2**: Cartesian coordinates of **MD2** chromophore.

| **Atom** | **X-axis** | **Y-axis** | **Z-axis** |
| --- | --- | --- | --- |
| C | -0.284777 | 1.30969 | 0.400459 |
| C | -1.353479 | 0.46936 | 0.011067 |
| C | -1.024231 | -0.83661 | -0.394847 |
| C | 0.284798 | -1.309716 | -0.400507 |
| C | 1.3535 | -0.469385 | -0.011116 |
| C | 1.024253 | 0.836585 | 0.394795 |
| H | -1.821483 | -1.480816 | -0.739047 |
| H | 1.821506 | 1.480792 | 0.738993 |
| C | 2.761432 | -0.877159 | -0.039327 |
| C | 3.85857 | -0.034188 | -0.063379 |
| S | 3.282453 | -2.57112 | 0.042339 |
| C | 5.090429 | -0.732607 | 0.004359 |
| H | 3.77106 | 1.041628 | -0.150829 |
| C | 4.939293 | -2.101491 | 0.078144 |
| C | -2.761411 | 0.877138 | 0.039274 |
| C | -3.858554 | 0.034172 | 0.06327 |
| S | -3.282423 | 2.571107 | -0.042294 |
| C | -5.09041 | 0.732599 | -0.004451 |
| H | -3.771052 | -1.04165 | 0.150646 |
| C | -4.939265 | 2.101486 | -0.07817 |
| C | -8.758716 | -0.261977 | 0.022123 |
| C | -7.408041 | -0.641424 | 0.05152 |
| C | -6.527826 | 0.448272 | 0.003637 |
| H | -7.115222 | -1.684244 | 0.105091 |
| C | 6.52784 | -0.44827 | -0.003753 |
| C | 7.199084 | -1.665955 | 0.061613 |
| C | 8.758722 | 0.261998 | -0.022325 |
| S | -8.906518 | 1.507585 | -0.06738 |
| S | 8.906542 | -1.50756 | 0.067261 |
| H | 7.115218 | 1.68425 | -0.105306 |
| C | 7.408044 | 0.641434 | -0.051691 |
| C | -7.199059 | 1.665965 | -0.061696 |
| C | -6.258796 | 2.865801 | -0.116773 |
| C | 6.25883 | -2.865795 | 0.116768 |
| C | -6.433224 | 3.668559 | -1.42606 |
| H | -7.426289 | 4.127421 | -1.466689 |
| H | -5.686754 | 4.467194 | -1.480875 |
| H | -6.312273 | 3.02404 | -2.300146 |
| C | -6.436389 | 3.788868 | 1.110386 |
| H | -5.686879 | 4.586324 | 1.092803 |
| H | -7.427628 | 4.25323 | 1.102327 |
| H | -6.321861 | 3.229516 | 2.042095 |
| C | 6.436413 | -3.788925 | -1.110346 |
| H | 7.427649 | -4.253293 | -1.102268 |
| H | 5.686899 | -4.586377 | -1.092718 |
| H | 6.321883 | -3.22962 | -2.042082 |
| C | 6.433285 | -3.668481 | 1.426094 |
| H | 5.686822 | -4.467118 | 1.480965 |
| H | 7.426354 | -4.127332 | 1.466733 |
| H | 6.312343 | -3.023916 | 2.300149 |
| C | -9.788086 | -1.243606 | 0.058967 |
| C | -11.161302 | -1.197421 | 0.046506 |
| H | -9.354786 | -2.237466 | 0.107412 |
| C | 9.788087 | 1.243633 | -0.05913 |
| C | 11.161302 | 1.19744 | -0.046619 |
| H | 9.354788 | 2.237489 | -0.107629 |
| C | -12.062245 | -2.362204 | 0.090473 |
| C | -11.773135 | -3.713207 | 0.139032 |
| C | -12.011077 | 0.025386 | -0.006245 |
| C | 12.011063 | -0.025374 | 0.006188 |
| C | 12.062251 | 2.362211 | -0.090631 |
| C | 11.773163 | 3.713216 | -0.139335 |
| O | -11.643464 | 1.193607 | -0.055589 |
| O | 11.64343 | -1.19359 | 0.055525 |
| C | -13.421515 | -1.819582 | 0.069608 |
| C | -13.408581 | -0.446604 | 0.013818 |
| C | 13.421518 | 1.819582 | -0.069631 |
| C | 13.408572 | 0.446604 | -0.01382 |
| C | -12.813793 | -4.690742 | 0.176234 |
| N | -13.659004 | -5.492237 | 0.207699 |
| C | -10.452039 | -4.253137 | 0.154253 |
| N | -9.383828 | -4.718652 | 0.166875 |
| C | 10.452076 | 4.25316 | -0.154774 |
| N | 9.383869 | 4.718678 | -0.167571 |
| C | 12.813829 | 4.690739 | -0.17645 |
| N | 13.65886 | 5.492466 | -0.206802 |
| O | -0.623754 | 2.576221 | 0.786161 |
| O | 0.623777 | -2.576246 | -0.786208 |
| C | 0.398264 | 3.480213 | 1.177015 |
| H | 0.932399 | 3.122797 | 2.066035 |
| H | 1.117156 | 3.651262 | 0.366122 |
| H | -0.107237 | 4.416806 | 1.414339 |
| C | -0.398242 | -3.48025 | -1.177033 |
| H | -1.117128 | -3.65128 | -0.366131 |
| H | -0.932384 | -3.122855 | -2.066058 |
| H | 0.107259 | -4.416847 | -1.41434 |
| S | -15.03404 | -2.484704 | 0.0974 |
| S | 15.034047 | 2.484698 | -0.097293 |
| C | -17.059288 | -0.513238 | 0.022028 |
| C | -15.706568 | -0.849405 | 0.032655 |
| C | -14.70166 | 0.151974 | -0.008957 |
| C | -15.069243 | 1.505695 | -0.062107 |
| C | -16.41442 | 1.841546 | -0.072546 |
| C | -17.408945 | 0.833359 | -0.030443 |
| H | -17.836774 | -1.267607 | 0.05357 |
| H | -14.305988 | 2.273931 | -0.093993 |
| C | 15.069198 | -1.505701 | 0.062197 |
| C | 16.414371 | -1.841564 | 0.072716 |
| C | 17.408908 | -0.833389 | 0.030662 |
| C | 17.059268 | 0.51321 | -0.021829 |
| C | 15.706552 | 0.849394 | -0.032532 |
| C | 14.701634 | -0.151977 | 0.009028 |
| H | 14.305935 | -2.273929 | 0.094055 |
| H | 17.836762 | 1.267573 | -0.053323 |
| Cl | -19.103251 | 1.244952 | -0.042779 |
| Cl | -16.855325 | 3.52899 | -0.138543 |
| Cl | 19.103211 | -1.244997 | 0.043094 |
| Cl | 16.855257 | -3.529013 | 0.138747 |

**Table S3**: Cartesian coordinates of **MD3** chromophore.

| **Atom** | **X-axis** | **Y-axis** | **Z-axis** |
| --- | --- | --- | --- |
| C | 0.303838 | 1.305172 | -0.401637 |
| C | 1.359656 | 0.449854 | -0.009593 |
| C | 1.011868 | -0.850561 | 0.398314 |
| C | -0.303817 | -1.304947 | 0.403374 |
| ZC | -1.359639 | -0.449639 | 0.011316 |
| C | -1.011843 | 0.850781 | -0.396588 |
| H | 1.799419 | -1.505475 | 0.744681 |
| H | -1.799381 | 1.505705 | -0.742962 |
| C | -2.773196 | -0.83755 | 0.039365 |
| C | -3.858403 | 0.020443 | 0.063892 |
| S | -3.317012 | -2.524522 | -0.042263 |
| C | -5.099413 | -0.661745 | 0.003453 |
| H | -3.756215 | 1.094911 | 0.151466 |
| C | -4.966924 | -2.032742 | -0.077593 |
| C | 2.773216 | 0.837744 | -0.03769 |
| C | 3.858389 | -0.02027 | -0.06271 |
| S | 3.317075 | 2.524664 | 0.044597 |
| C | 5.099426 | 0.661862 | 0.004753 |
| H | 3.756141 | -1.094697 | -0.150733 |
| C | 4.966983 | 2.03283 | 0.0795 |
| C | 8.755224 | -0.377257 | -0.027165 |
| C | 7.397917 | -0.740668 | -0.054938 |
| C | 6.532267 | 0.358295 | -0.005036 |
| H | 7.092576 | -1.779869 | -0.108726 |
| C | -6.532264 | -0.35821 | 0.005969 |
| C | -7.220301 | -1.568801 | -0.059174 |
| C | -8.755245 | 0.377292 | 0.027366 |
| S | 8.924135 | 1.391371 | 0.063264 |
| S | -8.924092 | -1.391383 | -0.062282 |
| H | -7.092649 | 1.779986 | 0.10859 |
| C | -7.397953 | 0.740751 | 0.055225 |
| C | 7.220347 | 1.568838 | 0.06052 |
| C | 6.295979 | 2.780488 | 0.118104 |
| C | -6.295894 | -2.780452 | -0.11607 |
| C | 6.482978 | 3.578334 | 1.428928 |
| H | 7.481639 | 4.024734 | 1.468828 |
| H | 5.746752 | 4.386138 | 1.485499 |
| H | 6.35458 | 2.934241 | 2.30225 |
| C | 6.485656 | 3.703136 | -1.107766 |
| H | 5.746243 | 4.509743 | -1.087975 |
| H | 7.48242 | 4.1553 | -1.098724 |
| H | 6.363592 | 3.147429 | -2.040688 |
| C | -6.485741 | -3.702546 | 1.11019 |
| H | -7.4825 | -4.154721 | 1.101209 |
| H | -5.746319 | -4.509158 | 1.090878 |
| H | -6.363818 | -3.146414 | 2.042878 |
| C | -6.482668 | -3.578898 | -1.426561 |
| H | -5.746397 | -4.386694 | -1.482662 |
| H | -7.481303 | -4.025361 | -1.466406 |
| H | -6.35417 | -2.935196 | -2.300157 |
| C | 9.770409 | -1.368549 | -0.064333 |
| C | 11.147245 | -1.337938 | -0.052074 |
| H | 9.327024 | -2.358058 | -0.112295 |
| C | -9.770462 | 1.368575 | 0.063862 |
| C | -11.147297 | 1.337926 | 0.051338 |
| H | -9.327113 | 2.358119 | 0.111438 |
| C | 12.032011 | -2.511495 | -0.094351 |
| C | 11.733975 | -3.86 | -0.142598 |
| C | 12.007036 | -0.124768 | -0.000816 |
| C | -12.007053 | 0.124713 | 0.000504 |
| C | -12.032095 | 2.511485 | 0.092854 |
| C | -11.734095 | 3.860019 | 0.140522 |
| O | 11.656851 | 1.048162 | 0.043232 |
| O | -11.656836 | -1.04823 | -0.04293 |
| C | 13.401824 | -1.984112 | -0.072256 |
| C | 13.401968 | -0.612382 | -0.017267 |
| C | -13.401894 | 1.984067 | 0.070681 |
| C | -13.401997 | 0.612309 | 0.016363 |
| C | 12.76966 | -4.842622 | -0.176801 |
| N | 13.61606 | -5.642954 | -0.204304 |
| C | 10.408304 | -4.388552 | -0.160036 |
| N | 9.334874 | -4.841352 | -0.174337 |
| C | -10.408437 | 4.388602 | 0.158021 |
| N | -9.335017 | 4.841424 | 0.172365 |
| C | -12.769805 | 4.842638 | 0.174006 |
| N | -13.616227 | 5.642967 | 0.200923 |
| O | 0.661695 | 2.565646 | -0.78882 |
| O | -0.661659 | -2.565418 | 0.790579 |
| C | -0.346254 | 3.483599 | -1.184943 |
| H | -0.883251 | 3.130933 | -2.074056 |
| H | -1.064117 | 3.667641 | -0.376059 |
| H | 0.173572 | 4.4118 | -1.42399 |
| C | 0.346293 | -3.483367 | 1.186703 |
| H | 1.064146 | -3.667421 | 0.377812 |
| H | 0.8833 | -3.130689 | 2.075804 |
| H | -0.173532 | -4.411564 | 1.425768 |
| S | 15.005607 | -2.667771 | -0.101548 |
| S | -15.005697 | 2.66771 | 0.099231 |
| C | 17.054041 | -0.725957 | -0.023166 |
| C | 15.695022 | -1.044698 | -0.040871 |
| C | 14.700965 | -0.030544 | 0.00536 |
| C | 15.084663 | 1.320961 | 0.048914 |
| C | 16.431042 | 1.62927 | 0.051084 |
| C | 17.410204 | 0.612428 | 0.031538 |
| H | 17.833582 | -1.478339 | -0.033916 |
| H | 14.341937 | 2.109401 | 0.060144 |
| C | -15.084645 | -1.321094 | -0.049302 |
| C | -16.431016 | -1.629434 | -0.051678 |
| C | -17.410203 | -0.612598 | -0.032893 |
| C | -17.054074 | 0.725817 | 0.021251 |
| C | -15.695065 | 1.044594 | 0.039164 |
| C | -14.700979 | 0.030439 | -0.006315 |
| H | -14.341904 | -2.109529 | -0.059949 |
| H | -17.833632 | 1.478191 | 0.031415 |
| N | -16.789504 | -3.056104 | 0.062572 |
| O | -17.695922 | -3.337568 | 0.838251 |
| O | -16.110389 | -3.851569 | -0.578616 |
| N | -18.844063 | -0.907897 | -0.204747 |
| O | -19.636209 | -0.174025 | 0.380067 |
| O | -19.134628 | -1.824794 | -0.964451 |
| N | 16.789543 | 3.055986 | -0.062537 |
| O | 17.695768 | 3.33781 | -0.838313 |
| O | 16.110621 | 3.851146 | 0.57923 |
| N | 18.844116 | 0.907628 | 0.203131 |
| O | 19.134902 | 1.824108 | 0.963255 |
| O | 19.636084 | 0.174073 | -0.382314 |

**Table S4**: Cartesian coordinates of **MD4** chromophore.

| **Atom** | **X-axis** | **Y-axis** | **Z-axis** |
| --- | --- | --- | --- |
| C | 0.326327 | -1.293243 | 0.423084 |
| C | 1.366916 | -0.427328 | 0.01422 |
| C | 0.996547 | 0.860455 | -0.413497 |
| C | -0.326387 | 1.2933 | -0.421793 |
| C | -1.366962 | 0.427371 | -0.012934 |
| C | -0.996616 | -0.860401 | 0.414779 |
| H | 1.772535 | 1.522026 | -0.773105 |
| H | -1.772631 | -1.521945 | 0.774387 |
| C | -2.786906 | 0.790996 | -0.043254 |
| C | -3.857488 | -0.085471 | -0.058002 |
| S | -3.359138 | 2.468911 | 0.024159 |
| C | -5.110045 | 0.575822 | 0.005336 |
| H | -3.736817 | -1.158812 | -0.135994 |
| C | -5.000633 | 1.949354 | 0.06722 |
| C | 2.786863 | -0.790957 | 0.044525 |
| C | 3.857486 | 0.085492 | 0.058315 |
| S | 3.359079 | -2.468954 | -0.021399 |
| C | 5.110018 | -0.575878 | -0.004539 |
| H | 3.7369 | 1.15891 | 0.135332 |
| C | 5.000573 | -1.949465 | -0.065114 |
| C | 8.747244 | 0.527598 | 0.012806 |
| C | 7.385077 | 0.867164 | 0.038715 |
| C | 6.537959 | -0.248013 | -0.000145 |
| H | 7.061547 | 1.901263 | 0.084106 |
| C | -6.537982 | 0.247942 | 0.000431 |
| C | -7.24597 | 1.445989 | 0.056305 |
| C | -8.74725 | -0.527689 | -0.013511 |
| S | 8.947303 | -1.237878 | -0.062573 |
| S | -8.947349 | 1.237709 | 0.063464 |
| H | -7.061525 | -1.901265 | -0.085887 |
| C | -7.385076 | -0.867213 | -0.039583 |
| C | 7.245921 | -1.446123 | -0.055006 |
| C | 6.342349 | -2.674192 | -0.098065 |
| C | -6.342424 | 2.674029 | 0.100682 |
| C | 6.540381 | -3.483333 | -1.400087 |
| H | 7.546659 | -3.912691 | -1.437028 |
| H | 5.818116 | -4.304366 | -1.446942 |
| H | 6.399723 | -2.850958 | -2.280073 |
| C | 6.54945 | -3.579611 | 1.137642 |
| H | 5.824852 | -4.399878 | 1.128524 |
| H | 7.554418 | -4.013438 | 1.132693 |
| H | 6.418606 | -3.015366 | 2.064238 |
| C | -6.549351 | 3.580696 | -1.134137 |
| H | -7.554298 | 4.014568 | -1.128864 |
| H | -5.824714 | 4.400917 | -1.124097 |
| H | -6.418424 | 3.017382 | -2.061288 |
| C | -6.540653 | 3.481856 | 1.403491 |
| H | -5.81845 | 4.302894 | 1.451247 |
| H | -7.546966 | 3.911106 | 1.440746 |
| H | -6.400052 | 2.848607 | 2.282857 |
| C | 9.746343 | 1.538455 | 0.044972 |
| C | 11.121587 | 1.531798 | 0.03594 |
| H | 9.284502 | 2.519728 | 0.08711 |
| C | -9.746339 | -1.538524 | -0.046616 |
| C | -11.121581 | -1.531858 | -0.037583 |
| H | -9.284498 | -2.519762 | -0.089559 |
| C | 11.98815 | 2.72082 | 0.078578 |
| C | 11.663809 | 4.063578 | 0.119208 |
| C | 12.004186 | 0.33289 | -0.009661 |
| C | -12.004144 | -0.332962 | 0.008988 |
| C | -11.988177 | -2.720825 | -0.081148 |
| C | -11.663885 | -4.063564 | -0.122787 |
| O | 11.669876 | -0.844943 | -0.057173 |
| O | -11.669796 | 0.844826 | 0.057338 |
| C | 13.364551 | 2.215826 | 0.066561 |
| C | 13.389026 | 0.843437 | 0.015061 |
| C | -13.364563 | -2.215798 | -0.068742 |
| C | -13.388997 | -0.843446 | -0.016207 |
| C | 12.678434 | 5.0681 | 0.156893 |
| N | 13.503395 | 5.890372 | 0.187687 |
| C | 10.32836 | 4.5675 | 0.124812 |
| N | 9.247336 | 5.00232 | 0.129464 |
| C | -10.328458 | -4.567537 | -0.128796 |
| N | -9.247458 | -5.002416 | -0.133773 |
| C | -12.678552 | -5.068013 | -0.161281 |
| N | -13.503554 | -5.89022 | -0.192695 |
| O | 0.705601 | -2.541501 | 0.829843 |
| O | -0.705697 | 2.541567 | -0.828493 |
| C | -0.286586 | -3.467645 | 1.245487 |
| H | -0.826032 | -3.107966 | 2.130356 |
| H | -1.004612 | -3.67675 | 0.442821 |
| H | 0.248251 | -4.383546 | 1.498624 |
| C | 0.286477 | 3.467723 | -1.244138 |
| H | 1.004532 | 3.676806 | -0.441491 |
| H | 0.825896 | 3.108074 | -2.129037 |
| H | -0.248368 | 4.383631 | -1.497231 |
| S | 14.957665 | 2.924719 | 0.101467 |
| S | -14.957698 | -2.924615 | -0.10416 |
| C | 17.03474 | 1.006367 | 0.045808 |
| C | 15.673277 | 1.310237 | 0.045954 |
| C | 14.699597 | 0.283872 | 0.000836 |
| C | 15.114012 | -1.056555 | -0.04855 |
| C | 16.466271 | -1.367075 | -0.04772 |
| C | 17.436878 | -0.325266 | -0.004933 |
| H | 17.781921 | 1.788964 | 0.081496 |
| H | 14.368445 | -1.840286 | -0.083868 |
| C | -15.113925 | 1.056555 | 0.048681 |
| C | -16.466176 | 1.367118 | 0.048075 |
| C | -17.436819 | 0.325362 | 0.004614 |
| C | -17.034715 | -1.006245 | -0.047068 |
| C | -15.67326 | -1.310153 | -0.047453 |
| C | -14.699549 | -0.28385 | -0.001622 |
| H | -14.368334 | 1.84024 | 0.084496 |
| H | -17.781914 | -1.788802 | -0.083296 |
| C | -18.931454 | 0.590104 | -0.054466 |
| C | -16.836122 | 2.835729 | 0.159515 |
| C | 16.836321 | -2.835726 | -0.158181 |
| C | 18.931514 | -0.589947 | 0.054395 |
| F | -19.307611 | 1.026304 | -1.273332 |
| F | -19.326529 | 1.503669 | 0.85107 |
| F | -19.634794 | -0.538938 | 0.192144 |
| F | -17.802939 | 3.189002 | -0.708967 |
| F | -17.264491 | 3.144663 | 1.400477 |
| F | -15.769659 | 3.625471 | -0.095661 |
| F | 17.802984 | -3.188387 | 0.71071 |
| F | 15.769854 | -3.62536 | 0.097297 |
| F | 17.264913 | -3.145428 | -1.398879 |
| F | 19.307582 | -1.02547 | 1.27352 |
| F | 19.326688 | -1.503989 | -0.85064 |
| F | 19.634832 | 0.538981 | -0.192777 |

**Table S5**: Cartesian coordinates of **MD5** chromophore.

| **Atom** | **X-axis** | **Y-axis** | **Z-axis** |
| --- | --- | --- | --- |
| C | -0.342751 | 1.257661 | 0.439297 |
| C | -1.382192 | 0.38624 | 0.03935 |
| C | -1.008772 | -0.904083 | -0.37877 |
| C | 0.315163 | -1.33359 | -0.386364 |
| C | 1.35437 | -0.461429 | 0.012498 |
| C | 0.981439 | 0.828498 | 0.430926 |
| H | -1.783361 | -1.570916 | -0.73145 |
| H | 1.756894 | 1.494791 | 0.782997 |
| C | 2.77547 | -0.820133 | -0.020373 |
| C | 3.841851 | 0.060631 | -0.057926 |
| S | 3.355576 | -2.493803 | 0.074637 |
| C | 5.097838 | -0.593694 | 0.009741 |
| H | 3.715282 | 1.131719 | -0.155604 |
| C | 4.99525 | -1.96624 | 0.098246 |
| C | -2.802998 | 0.746632 | 0.068301 |
| C | -3.871943 | -0.132255 | 0.073951 |
| S | -3.379652 | 2.423695 | 0.0129 |
| C | -5.125973 | 0.52611 | 0.012959 |
| H | -3.749628 | -1.20597 | 0.143416 |
| C | -5.019932 | 1.900256 | -0.037159 |
| C | -8.75911 | -0.590449 | 0.0065 |
| C | -7.396887 | -0.92499 | 0.035249 |
| C | -6.553208 | 0.194224 | 0.009934 |
| H | -7.069648 | -1.958267 | 0.071881 |
| C | 6.524176 | -0.259356 | -0.012094 |
| C | 7.238325 | -1.452157 | 0.060902 |
| C | 8.728643 | 0.527732 | -0.058475 |
| S | -8.965733 | 1.17441 | -0.053315 |
| S | 8.939126 | -1.234428 | 0.050016 |
| H | 7.035678 | 1.8909 | -0.142884 |
| C | 7.36518 | 0.859622 | -0.079781 |
| C | -7.264218 | 1.389671 | -0.037611 |
| C | -6.364034 | 2.620885 | -0.068377 |
| C | 6.341037 | -2.683521 | 0.135711 |
| C | -6.560168 | 3.439582 | -1.364571 |
| H | -7.567675 | 3.866217 | -1.401393 |
| H | -5.840066 | 4.263021 | -1.403449 |
| H | -6.415364 | 2.814135 | -2.248824 |
| C | -6.576963 | 3.516038 | 1.173627 |
| H | -5.8554 | 4.339138 | 1.173153 |
| H | -7.583663 | 3.946011 | 1.169804 |
| H | -6.446507 | 2.944779 | 2.095974 |
| C | 6.542962 | -3.61358 | -1.082327 |
| H | 7.549942 | -4.042683 | -1.075992 |
| H | 5.822141 | -4.436651 | -1.050693 |
| H | 6.402813 | -3.069216 | -2.019385 |
| C | 6.552525 | -3.464622 | 1.452595 |
| H | 5.835611 | -4.288861 | 1.521353 |
| H | 7.561676 | -3.887045 | 1.491377 |
| H | 6.41406 | -2.814917 | 2.320247 |
| C | -9.755077 | -1.607166 | 0.021076 |
| C | -11.128685 | -1.607403 | 0.002906 |
| H | -9.287981 | -2.586246 | 0.053451 |
| C | 9.72229 | 1.54419 | -0.11815 |
| C | 11.096567 | 1.545743 | -0.123195 |
| H | 9.253868 | 2.52166 | -0.173387 |
| C | -11.990156 | -2.8024 | 0.019963 |
| C | -11.655469 | -4.143239 | 0.046524 |
| C | -12.018908 | -0.412509 | -0.031277 |
| C | 11.987713 | 0.352493 | -0.070386 |
| C | 11.956068 | 2.739045 | -0.194828 |
| C | 11.621484 | 4.079421 | -0.243892 |
| O | -11.687541 | 0.767091 | -0.058079 |
| O | 11.658733 | -0.824883 | 0.006271 |
| C | -13.366844 | -2.304921 | -0.000229 |
| C | -13.400416 | -0.930933 | -0.02717 |
| C | 13.333299 | 2.241516 | -0.199089 |
| C | 13.36952 | 0.870496 | -0.12781 |
| C | -12.660938 | -5.157659 | 0.061021 |
| N | -13.474926 | -5.991267 | 0.073735 |
| C | -10.316455 | -4.637475 | 0.061195 |
| N | -9.232849 | -5.065945 | 0.073172 |
| C | 10.282909 | 4.574707 | -0.231422 |
| N | 9.199664 | 5.004108 | -0.221214 |
| C | 12.627371 | 5.091504 | -0.306575 |
| N | 13.443166 | 5.922043 | -0.355966 |
| O | -0.723891 | 2.508243 | 0.837838 |
| O | 0.697036 | -2.584329 | -0.783276 |
| C | 0.267439 | 3.440753 | 1.240421 |
| H | 0.81203 | 3.090835 | 2.126092 |
| H | 0.981394 | 3.644425 | 0.432709 |
| H | -0.26876 | 4.357541 | 1.487539 |
| C | -0.293757 | -3.516186 | -1.188979 |
| H | -1.009672 | -3.720361 | -0.383114 |
| H | -0.836126 | -3.165284 | -2.075621 |
| H | 0.242691 | -4.432923 | -1.435671 |
| S | -14.95584 | -3.023637 | -0.002295 |
| S | 14.922103 | 2.961457 | -0.272251 |
| C | -17.048136 | -1.121895 | -0.052504 |
| C | -15.683934 | -1.413341 | -0.038794 |
| C | -14.713764 | -0.377072 | -0.047711 |
| C | -15.132718 | 0.962636 | -0.090782 |
| C | -16.491567 | 1.258612 | -0.110372 |
| C | -17.451869 | 0.210583 | -0.06605 |
| H | -17.800292 | -1.903093 | -0.042868 |
| H | -14.394785 | 1.754351 | -0.129229 |
| C | 15.110706 | -1.016197 | -0.071279 |
| C | 16.471567 | -1.309058 | -0.079385 |
| C | 17.432212 | -0.264952 | -0.164162 |
| C | 17.018436 | 1.065738 | -0.228535 |
| C | 15.653123 | 1.354056 | -0.203903 |
| C | 14.684644 | 0.317614 | -0.128334 |
| H | 14.389669 | -1.824043 | -0.021274 |
| H | 17.75359 | 1.859571 | -0.317176 |
| C | 18.916851 | -0.516036 | -0.344666 |
| C | 16.836364 | -2.756889 | 0.00446 |
| C | -18.934118 | 0.447354 | 0.004632 |
| C | -16.931554 | 2.672306 | -0.330217 |
| O | 19.725832 | -0.400072 | 0.731252 |
| O | 19.387743 | -0.711927 | -1.437473 |
| O | 18.131156 | -2.934613 | 0.341371 |
| O | 16.05961 | -3.66995 | -0.17982 |
| O | -19.249333 | 1.341315 | 0.958545 |
| O | -19.743922 | -0.166541 | -0.656122 |
| O | -17.947267 | 2.994089 | -0.911135 |
| O | -16.034085 | 3.558224 | 0.150319 |
| C | -16.345108 | 4.940106 | -0.099094 |
| H | -15.526581 | 5.507731 | 0.342001 |
| H | -16.413021 | 5.130805 | -1.172751 |
| H | -17.295475 | 5.207468 | 0.368388 |
| C | -20.644369 | 1.682267 | 1.040926 |
| H | -20.960181 | 2.183759 | 0.123122 |
| H | -21.251443 | 0.786955 | 1.190711 |
| H | -20.731048 | 2.355209 | 1.893317 |
| C | 18.59 | -4.298942 | 0.373482 |
| H | 18.048429 | -4.863166 | 1.136031 |
| H | 18.436828 | -4.771946 | -0.598537 |
| H | 19.651178 | -4.242585 | 0.611137 |
| C | 19.171455 | -0.320396 | 2.052767 |
| H | 18.573005 | 0.584897 | 2.187463 |
| H | 18.565081 | -1.202202 | 2.273171 |
| H | 20.030028 | -0.289308 | 2.724049 |

**Table S6**: Cartesian coordinates of **MD6** chromophore.

| **Atom** | **X-axis** | **Y-axis** | **Z-axis** |
| --- | --- | --- | --- |
| C | -0.278191 | -1.31464 | -0.394149 |
| C | -1.350933 | -0.477732 | -0.008291 |
| C | -1.028664 | 0.831728 | 0.391695 |
| C | 0.277937 | 1.311508 | 0.394984 |
| C | 1.350685 | 0.474637 | 0.009087 |
| C | 1.028424 | -0.83485 | -0.390875 |
| H | -1.828971 | 1.473611 | 0.733085 |
| H | 1.82873 | -1.476747 | -0.732241 |
| C | 2.756339 | 0.890422 | 0.035139 |
| C | 3.858367 | 0.054035 | 0.059606 |
| S | 3.266914 | 2.587685 | -0.048565 |
| C | 5.085659 | 0.760327 | -0.009543 |
| H | 3.777681 | -1.022164 | 0.148276 |
| C | 4.926205 | 2.128335 | -0.084537 |
| C | -2.756633 | -0.893357 | -0.034315 |
| C | -3.858453 | -0.056726 | -0.058891 |
| S | -3.267587 | -2.590486 | 0.049643 |
| C | -5.085891 | -0.762717 | 0.010343 |
| H | -3.777506 | 1.01944 | -0.14771 |
| C | -4.9268 | -2.130746 | 0.085561 |
| C | -8.760844 | 0.206527 | -0.01668 |
| C | -7.411068 | 0.595563 | -0.046492 |
| C | -6.524442 | -0.486931 | 0.002433 |
| H | -7.125528 | 1.640327 | -0.101239 |
| C | 6.524307 | 0.484947 | -0.00175 |
| C | 7.188351 | 1.708401 | -0.06854 |
| C | 8.760962 | -0.20777 | 0.017142 |
| S | -8.896014 | -1.56478 | 0.074661 |
| S | 8.895529 | 1.563598 | -0.073963 |
| H | 7.126125 | -1.642145 | 0.101533 |
| C | 7.411311 | -0.597272 | 0.046957 |
| C | -7.188861 | -1.710158 | 0.069397 |
| C | -6.24118 | -2.903768 | 0.125793 |
| C | 6.24034 | 2.901737 | -0.124676 |
| C | -6.410791 | -3.705569 | 1.436531 |
| H | -7.40043 | -4.171587 | 1.477559 |
| H | -5.658761 | -4.498772 | 1.491943 |
| H | -6.294048 | -3.059347 | 2.30992 |
| C | -6.414279 | -3.829686 | -1.100093 |
| H | -5.659299 | -4.621773 | -1.081365 |
| H | -7.4021 | -4.30107 | -1.090104 |
| H | -6.304101 | -3.271398 | -2.032944 |
| C | 6.413224 | 3.827468 | 1.101386 |
| H | 7.400906 | 4.299146 | 1.091439 |
| H | 5.65801 | 4.619336 | 1.082854 |
| H | 6.303258 | 3.268966 | 2.034135 |
| C | 6.409662 | 3.703847 | -1.435257 |
| H | 5.657373 | 4.496815 | -1.490488 |
| H | 7.399149 | 4.170192 | -1.476218 |
| H | 6.293102 | 3.057763 | -2.308774 |
| C | -9.795027 | 1.178715 | -0.054151 |
| C | -11.170559 | 1.122691 | -0.042218 |
| H | -9.370141 | 2.17624 | -0.102768 |
| C | 9.795584 | -1.179495 | 0.054319 |
| C | 11.171083 | -1.122668 | 0.042139 |
| H | 9.37123 | -2.177257 | 0.102846 |
| C | -12.076841 | 2.280292 | -0.08615 |
| C | -11.80212 | 3.633803 | -0.134467 |
| C | -12.009102 | -0.105617 | 0.009482 |
| C | 12.008801 | 0.106214 | -0.009635 |
| C | 12.078167 | -2.279642 | 0.085815 |
| C | 11.804547 | -3.633387 | 0.134089 |
| O | -11.6372 | -1.271969 | 0.058442 |
| O | 11.636104 | 1.272324 | -0.058364 |
| C | -13.436178 | 1.728229 | -0.065798 |
| C | -13.412095 | 0.356789 | -0.01102 |
| C | 13.437123 | -1.726651 | 0.065164 |
| C | 13.412106 | -0.355232 | 0.010473 |
| C | -12.854002 | 4.599023 | -0.171468 |
| N | -13.712231 | 5.386549 | -0.201659 |
| C | -10.485936 | 4.185669 | -0.149771 |
| N | -9.420914 | 4.657988 | -0.162392 |
| C | 10.488811 | -4.186325 | 0.14961 |
| N | 9.424165 | -4.659487 | 0.162417 |
| C | 12.857353 | -4.59764 | 0.170735 |
| N | 13.716519 | -5.384154 | 0.20055 |
| O | -0.611052 | -2.584309 | -0.773586 |
| O | 0.610784 | 2.581166 | 0.774482 |
| C | 0.414976 | -3.485843 | -1.160733 |
| H | 0.946845 | -3.130077 | -2.05169 |
| H | 1.134642 | -3.649762 | -0.349133 |
| H | -0.086569 | -4.425663 | -1.393378 |
| C | -0.415237 | 3.482715 | 1.161613 |
| H | -1.134885 | 3.646651 | 0.349998 |
| H | -0.947132 | 3.126949 | 2.052555 |
| H | 0.086313 | 4.422529 | 1.394277 |
| S | -15.052454 | 2.383007 | -0.09358 |
| S | 15.053853 | -2.380339 | 0.092517 |
| C | -17.065216 | 0.405543 | -0.02073 |
| C | -15.713935 | 0.746663 | -0.030471 |
| C | -14.700536 | -0.250053 | 0.010641 |
| C | -15.056518 | -1.606251 | 0.062038 |
| C | -16.40404 | -1.957134 | 0.071609 |
| C | -17.414203 | -0.946762 | 0.030157 |
| H | -17.843514 | 1.1596 | -0.051777 |
| H | -14.284865 | -2.366517 | 0.093243 |
| C | 15.055216 | 1.608937 | -0.062872 |
| C | 16.402497 | 1.960731 | -0.072766 |
| C | 17.413352 | 0.951029 | -0.03162 |
| C | 17.065284 | -0.401513 | 0.019278 |
| C | 15.714233 | -0.743549 | 0.029338 |
| C | 14.70016 | 0.252494 | -0.011462 |
| H | 14.283037 | 2.368681 | -0.093843 |
| H | 17.844094 | -1.155049 | 0.0501 |
| C | 18.799531 | 1.309278 | -0.041851 |
| N | 19.932441 | 1.571979 | -0.049091 |
| C | 16.765468 | 3.346115 | -0.124634 |
| N | 17.035051 | 4.476295 | -0.167035 |
| C | -18.800622 | -1.304084 | 0.040062 |
| N | -19.933707 | -1.566027 | 0.047032 |
| C | -16.767966 | -3.34227 | 0.123459 |
| N | -17.038329 | -4.472263 | 0.165848 |

**Table S7**: Cartesian coordinates of **MD7** chromophore.

| **Atom** | **X-axis** | **Y-axis** | **Z-axis** |
| --- | --- | --- | --- |
| C | -0.16764 | -1.340537 | -0.359026 |
| C | -1.308467 | -0.585451 | -0.000955 |
| C | -1.095355 | 0.755843 | 0.366159 |
| C | 0.167499 | 1.340967 | 0.363303 |
| C | 1.30832 | 0.585876 | 0.005196 |
| C | 1.095189 | -0.755405 | -0.361921 |
| H | -1.947222 | 1.338763 | 0.68742 |
| H | 1.947039 | -1.33832 | -0.683229 |
| C | 2.675536 | 1.114504 | 0.024812 |
| C | 3.841963 | 0.369998 | 0.052326 |
| S | 3.048826 | 2.846741 | -0.071324 |
| C | 5.008688 | 1.17121 | -0.024797 |
| H | 3.849327 | -0.708608 | 0.147911 |
| C | 4.740204 | 2.521247 | -0.107389 |
| C | -2.675635 | -1.114194 | -0.020753 |
| C | -3.842129 | -0.369816 | -0.048847 |
| S | -3.048777 | -2.846441 | 0.075765 |
| C | -5.008794 | -1.171136 | 0.028002 |
| H | -3.849566 | 0.708761 | -0.144762 |
| C | -4.740207 | -2.521116 | 0.111105 |
| C | -8.74826 | -0.49227 | 0.010414 |
| C | -7.43554 | 0.000002 | -0.027392 |
| C | -6.465413 | -1.010994 | 0.024528 |
| H | -7.232655 | 1.063467 | -0.090292 |
| C | 6.4653 | 1.010909 | -0.022138 |
| C | 7.029804 | 2.280303 | -0.09903 |
| C | 8.748104 | 0.491947 | -0.009641 |
| S | -8.74535 | -2.266999 | 0.113432 |
| S | 8.745312 | 2.266724 | -0.111882 |
| H | 7.232405 | -1.063681 | 0.091339 |
| C | 7.43536 | -0.000208 | 0.028827 |
| C | -7.029829 | -2.280419 | 0.101548 |
| C | -5.989704 | -3.394979 | 0.159339 |
| C | 5.98977 | 3.394989 | -0.155919 |
| C | -6.088478 | -4.204251 | 1.472307 |
| H | -7.037021 | -4.748835 | 1.518543 |
| H | -5.274246 | -4.933684 | 1.527621 |
| H | -6.022191 | -3.547721 | 2.343303 |
| C | -6.092301 | -4.335214 | -1.063372 |
| H | -5.277505 | -5.065967 | -1.046289 |
| H | -7.040426 | -4.882183 | -1.049381 |
| H | -6.029874 | -3.771653 | -1.997478 |
| C | 6.093059 | 4.334885 | 1.066987 |
| H | 7.041258 | 4.88172 | 1.052706 |
| H | 5.278362 | 5.065763 | 1.050468 |
| H | 6.030979 | 3.771087 | 2.000973 |
| C | 6.088002 | 4.204605 | -1.468725 |
| H | 5.273816 | 4.934135 | -1.523457 |
| H | 7.036579 | 4.749105 | -1.515265 |
| H | 6.021236 | 3.548311 | -2.339864 |
| C | -9.857812 | 0.39949 | -0.031542 |
| C | -11.221384 | 0.239358 | -0.014752 |
| H | -9.509275 | 1.425694 | -0.089632 |
| C | 9.857636 | -0.399904 | 0.031005 |
| C | 11.22119 | -0.239778 | 0.012942 |
| H | 9.509108 | -1.42612 | 0.088915 |
| C | -12.21498 | 1.327446 | -0.068181 |
| C | -12.034839 | 2.697247 | -0.136321 |
| C | -11.964886 | -1.052349 | 0.055384 |
| C | 11.964563 | 1.051992 | -0.05724 |
| C | 12.214921 | -1.327872 | 0.064539 |
| C | 12.034771 | -2.697707 | 0.131757 |
| O | -11.49124 | -2.179248 | 0.114968 |
| O | 11.490811 | 2.17891 | -0.11559 |
| C | -13.51978 | 0.675696 | -0.033455 |
| C | -13.402044 | -0.70008 | 0.038139 |
| C | 13.519661 | -0.676029 | 0.028792 |
| C | 13.401742 | 0.699789 | -0.041759 |
| C | -13.156125 | 3.57947 | -0.180551 |
| N | -14.071611 | 4.299771 | -0.216662 |
| C | -10.763523 | 3.343994 | -0.167061 |
| N | -9.736867 | 3.89509 | -0.192624 |
| C | 10.763524 | -3.344544 | 0.163356 |
| N | 9.737025 | -3.895895 | 0.189582 |
| C | 13.156418 | -3.580094 | 0.173424 |
| N | 14.074578 | -4.297009 | 0.207281 |
| O | -0.391373 | -2.64328 | -0.706756 |
| O | 0.391246 | 2.64371 | 0.711004 |
| C | 0.710424 | -3.469751 | -1.049141 |
| H | 1.223649 | -3.10422 | -1.947146 |
| H | 1.430253 | -3.543408 | -0.224569 |
| H | 0.29144 | -4.455895 | -1.251857 |
| C | -0.710555 | 3.470198 | 1.053333 |
| H | -1.430369 | 3.543813 | 0.228745 |
| H | -1.223797 | 3.104706 | 1.951344 |
| H | -0.291577 | 4.456352 | 1.256011 |
| S | -15.18158 | 1.163905 | -0.05729 |
| S | 15.181596 | -1.164057 | 0.05048 |
| C | -15.685623 | -0.503259 | 0.029761 |
| C | -14.637137 | -1.388075 | 0.075079 |
| C | 15.685321 | 0.503211 | -0.036114 |
| C | 14.636706 | 1.387957 | -0.079614 |
| H | 16.743854 | 0.7271 | -0.049591 |
| H | 14.745046 | 2.463651 | -0.135144 |
| H | -14.745695 | -2.46372 | 0.131185 |
| H | -16.744189 | -0.727036 | 0.042151 |

**Table S8**: Cartesian coordinates of **MD8** chromophore.

| **Atom** | **X-axis** | **Y-axis** | **Z-axis** |
| --- | --- | --- | --- |
| C | 0.220168 | 1.329071 | -0.375731 |
| C | 1.330523 | 0.533019 | -0.010581 |
| C | 1.065065 | -0.794792 | 0.371006 |
| C | -0.220164 | -1.328981 | 0.375873 |
| C | -1.330519 | -0.53293 | 0.010723 |
| C | -1.06506 | 0.794884 | -0.370856 |
| H | 1.893769 | -1.407811 | 0.69702 |
| H | -1.893766 | 1.407908 | -0.696857 |
| C | -2.717487 | -1.007033 | 0.037469 |
| C | -3.853483 | -0.216957 | 0.06552 |
| S | -3.158887 | -2.723374 | -0.050676 |
| C | -5.051239 | -0.971789 | -0.004862 |
| H | -3.817586 | 0.861471 | 0.156847 |
| C | -4.836225 | -2.33166 | -0.08329 |
| C | 2.717491 | 1.007115 | -0.03734 |
| C | 3.85348 | 0.217029 | -0.065432 |
| S | 3.158905 | 2.723447 | 0.050868 |
| C | 5.051243 | 0.97185 | 0.004955 |
| H | 3.817569 | -0.861395 | -0.156794 |
| C | 4.836241 | 2.33172 | 0.083434 |
| C | 8.761008 | 0.145985 | -0.016559 |
| C | 7.430255 | -0.294348 | -0.050815 |
| C | 6.500574 | 0.754569 | -0.00152 |
| H | 7.185448 | -1.349229 | -0.108883 |
| C | -6.500573 | -0.754521 | 0.001581 |
| C | -7.11426 | -2.00087 | -0.069191 |
| C | -8.761013 | -0.145957 | 0.016557 |
| S | 8.827954 | 1.919839 | 0.079162 |
| S | -8.827941 | -1.919816 | -0.079085 |
| H | -7.185469 | 1.349275 | 0.108883 |
| C | -7.430264 | 0.29439 | 0.050834 |
| C | 7.114274 | 2.00091 | 0.069287 |
| C | 6.119215 | 3.155849 | 0.125585 |
| C | -6.119191 | -3.155803 | -0.125433 |
| C | 6.253458 | 3.964173 | 1.436052 |
| H | 7.223636 | 4.469526 | 1.478874 |
| H | 5.469848 | 4.726467 | 1.491019 |
| H | 6.162232 | 3.313235 | 2.308991 |
| C | 6.255151 | 4.08809 | -1.099927 |
| H | 5.469554 | 4.85013 | -1.08257 |
| H | 7.223989 | 4.597581 | -1.09006 |
| H | 6.168015 | 3.525033 | -2.032369 |
| C | -6.255137 | -4.088001 | 1.100112 |
| H | -7.22397 | -4.5975 | 1.090249 |
| H | -5.469533 | -4.850034 | 1.082792 |
| H | -6.168019 | -3.524909 | 2.032534 |
| C | -6.253406 | -3.964175 | -1.435872 |
| H | -5.469787 | -4.726463 | -1.4908 |
| H | -7.223578 | -4.46954 | -1.478691 |
| H | -6.162173 | -3.313267 | -2.308834 |
| C | 9.835485 | -0.788743 | -0.055272 |
| C | 11.203567 | -0.680861 | -0.03822 |
| H | 9.447639 | -1.800768 | -0.109876 |
| C | -9.835502 | 0.788758 | 0.05522 |
| C | -11.20358 | 0.68085 | 0.038162 |
| H | -9.447672 | 1.800792 | 0.109776 |
| C | 12.158153 | -1.806988 | -0.086439 |
| C | 11.920051 | -3.169494 | -0.148016 |
| C | 11.994511 | 0.579604 | 0.027378 |
| C | -11.994498 | -0.579635 | -0.027367 |
| C | -12.158188 | 1.806959 | 0.086334 |
| C | -11.920111 | 3.16947 | 0.147887 |
| O | 11.576136 | 1.729326 | 0.082272 |
| O | -11.576099 | -1.729353 | -0.082152 |
| C | 13.485742 | -1.209749 | -0.053891 |
| C | 13.412656 | 0.1715 | 0.012704 |
| C | -13.485765 | 1.209695 | 0.053761 |
| C | -13.412651 | -0.171556 | -0.012769 |
| C | 13.001302 | -4.100764 | -0.187151 |
| N | 13.884245 | -4.860909 | -0.219019 |
| C | 10.623286 | -3.763032 | -0.175533 |
| N | 9.575957 | -4.274164 | -0.198372 |
| C | -10.623357 | 3.763031 | 0.175418 |
| N | -9.576035 | 4.274176 | 0.198269 |
| C | -13.001379 | 4.100723 | 0.186973 |
| N | -13.884336 | 4.860851 | 0.218805 |
| O | 0.495503 | 2.617951 | -0.738017 |
| O | -0.495497 | -2.617858 | 0.738175 |
| C | -0.572471 | 3.483366 | -1.090893 |
| H | -1.100837 | 3.126912 | -1.983776 |
| H | -1.288076 | 3.596949 | -0.267149 |
| H | -0.114439 | 4.449141 | -1.306816 |
| C | 0.572513 | -3.483369 | 1.090711 |
| H | 1.288007 | -3.596764 | 0.266846 |
| H | 1.101001 | -3.127132 | 1.983609 |
| H | 0.114496 | -4.449187 | 1.306474 |
| S | 15.130189 | -1.800182 | -0.076484 |
| S | -15.130224 | 1.800097 | 0.076272 |
| C | 15.720189 | -0.149382 | 0.005323 |
| C | 14.678492 | 0.785051 | 0.046995 |
| C | -15.720191 | 0.149283 | -0.005485 |
| C | -14.678474 | -0.785132 | -0.047085 |
| C | 17.016011 | 0.435945 | 0.038074 |
| H | 17.950448 | -0.110298 | 0.014721 |
| C | -16.932355 | -1.801128 | -0.104191 |
| C | -17.016001 | -0.436072 | -0.03826 |
| H | -17.752793 | -2.504871 | -0.141059 |
| H | -17.95045 | 0.110154 | -0.014966 |
| S | 15.292926 | 2.409284 | 0.128043 |
| S | -15.292874 | -2.409381 | -0.128077 |
| C | 16.932394 | 1.801003 | 0.103998 |
| H | 17.752847 | 2.504732 | 0.140822 |

**Table S9:** Wave length, excitation energy and oscillator strength of investigated compound **MR1** in chloroform solvent at B3LYP/6-31G (d, p) level of theory.

| **NO** | **DFT λ (nm)** | **E(eV)** | ***f*** | **MO contributions** |
| --- | --- | --- | --- | --- |
| 1 | 738.221 | 1.680 | 0.250 | H→L (98%) |
| 2 | 731.168 | 1.696 | 0.000 | H→L+1 (99%) |
| 3 | 548.578 | 2.260 | 0.059 | H→L+2 (96%), H-1→L+3 (2%) |
| 4 | 547.609 | 2.264 | 0.000 | H→L+3 (92%), H-1→L (3%) |
| 5 | 532.304 | 2.329 | 0.793 | H-2→L (14%), H-1→L+1 (82%) |
| 6 | 530.777 | 2.336 | 0.000 | H-2→L+1 (10%), H-1→L (83%), H→L+3 (5%) |

**Table S10:** Wave length, excitation energy and oscillator strength of investigated compound **MD2** in chloroform solvent at B3LYP/6-31G (d, p) level of theory.

| **NO** | **DFT λ (nm)** | **E(eV)** | ***f*** | **MO contributions** |
| --- | --- | --- | --- | --- |
| 1 | 814.186 | 1.523 | 0.132 | H→L (98%) |
| 2 | 810.248 | 1.530 | 0.000 | H→L+1 (98%) |
| 3 | 598.408 | 2.072 | 0.000 | H-2→L+1 (17%), H-1→L (74%), H→L+3 (2%) |
| 4 | 598.264 | 2.072 | 0.247 | H-2→L (18%), H-1→L+1 (74%) |
| 5 | 561.167 | 2.209 | 0.326 | H→L+2 (94%), H-1→L+1 (2%) |
| 6 | 556.657 | 2.227 | 0.000 | H→L+3 (93%), H-1→L (4%) |

**Table S11:** Wave length, excitation energy and oscillator strength of investigated compound **MD3** in chloroform solvent at B3LYP/6-31G (d, p) level of theory.

| **NO** | **DFT λ (nm)** | **E(eV)** | ***f*** | **MO contributions** |
| --- | --- | --- | --- | --- |
| 1 | 939.844 | 1.319 | 0.134 | H→L (99%) |
| 2 | 933.757 | 1.328 | 0.000 | H→L+1 (99%) |
| 3 | 643.505 | 1.927 | 0.000 | H-2→L+1 (13%), H-1→L (71%), H→L+3 (10%) |
| 4 | 643.138 | 1.928 | 0.226 | H-2→L (14%), H-1→L+1 (72%), H→L+2 (8%) |
| 5 | 626.341 | 1.980 | 0.137 | H→L+2 (86%), H-1→L+1 (8%), H→L+4 (2%) |
| 6 | 625.299 | 1.983 | 0.000 | H-1→L (10%), H→L+3 (86%) |

**Table S12:** Wave length, excitation energy and oscillator strength of investigated compound **MD4** in chloroform solvent at B3LYP/6-31G (d, p) level of theory.

| **NO** | **DFT λ (nm)** | **E(eV)** | ***f*** | **MO contributions** |
| --- | --- | --- | --- | --- |
| 1 | 840.742 | 1.475 | 0.130 | H→L (98%) |
| 2 | 836.375 | 1.482 | 0.000 | H→L+1 (98%) |
| 3 | 605.865 | 2.046 | 0.001 | H-2→L+1 (16%), H-1→L (76%), H→L+3 (2%) |
| 4 | 605.687 | 2.047 | 0.250 | H-2→L (17%), H-1→L+1 (76%) |
| 5 | 567.745 | 2.184 | 0.294 | H→L+2 (95%), H-1→L+1 (2%) |
| 6 | 563.360 | 2.201 | 0.000 | H→L+3 (93%), H-1→L (4%) |

**Table S13:** Wave length, excitation energy and oscillator strength of investigated compound **MD5** in chloroform solvent at B3LYP/6-31G (d, p) level of theory.

| **NO** | **DFT λ (nm)** | **E(eV)** | ***f*** | **MO contributions** |
| --- | --- | --- | --- | --- |
| 1 | 819.893 | 1.512 | 0.115 | H→L (89%), H→L+1 (9%) |
| 2 | 814.453 | 1.522 | 0.017 | H→L+1 (89%), H→L (9%) |
| 3 | 600.146 | 2.066 | 0.103 | H-2→L (17%), H-1→L (75%) |
| 4 | 599.421 | 2.068 | 0.144 | H-2→L+1 (16%), H-1→L+1 (75%) |
| 5 | 562.083 | 2.206 | 0.318 | H→L+2 (89%), H→L+3 (5%) |
| 6 | 557.583 | 2.224 | 0.002 | H→L+3 (88%), H-1→L (3%), H→L+2 (5%) |

**Table S14:** Wave length, excitation energy and oscillator strength of investigated compound **MD6** in chloroform solvent at B3LYP/6-31G (d, p) level of theory.

| **NO** | **DFT λ (nm)** | **E(eV)** | ***f*** | **MO contributions** |
| --- | --- | --- | --- | --- |
| 1 | 906.649 | 1.368 | 0.131 | H→L (99%) |
| 2 | 901.244 | 1.376 | 0.000 | H→L+1 (99%) |
| 3 | 630.001 | 1.968 | 0.000 | H-2→L+1 (16%), H-1→L (78%) |
| 4 | 629.841 | 1.969 | 0.273 | H-2→L (17%), H-1→L+1 (78%) |
| 5 | 585.052 | 2.119 | 0.266 | H→L+2 (95%) |
| 6 | 580.668 | 2.135 | 0.000 | H→L+3 (92%), H-1→L (4%) |

**Table S15:** Wave length, excitation energy and oscillator strength of investigated compound **MD7** in chloroform solvent at B3LYP/6-31G (d, p) level of theory.

| **NO** | **DFT λ (nm)** | **E(eV)** | ***f*** | **MO contributions** |
| --- | --- | --- | --- | --- |
| 1 | 725.690 | 1.709 | 0.158 | H→L (98%) |
| 2 | 721.972 | 1.717 | 0.000 | H→L+1 (98%) |
| 3 | 547.682 | 2.264 | 0.000 | H-2→L+1 (14%), H-1→L (60%), H→L+3 (20%) |
| 4 | 547.126 | 2.266 | 0.143 | H-2→L (14%), H-1→L+1 (59%), H→L+2 (20%) |
| 5 | 536.728 | 2.310 | 0.482 | H-1→L+1 (19%), H→L+2 (76%) |
| 6 | 532.304 | 2.329 | 0.000 | H-1→L (20%), H→L+3 (76%) |

**Table S16:** Wave length, excitation energy and oscillator strength of investigated compound **MD8** in chloroform solvent at B3LYP/6-31G (d, p) level of theory.

| **NO** | **DFT λ (nm)** | **E(eV)** | ***f*** | **MO contributions** |
| --- | --- | --- | --- | --- |
| 1 | 744.158 | 1.666 | 0.141 | H→L (97%) |
| 2 | 741.045 | 1.673 | 0.000 | H→L+1 (98%) |
| 3 | 569.676 | 2.176 | 0.000 | H-2→L+1 (18%),H-1→L (66%), H-3→L+1 (2%), H→L+3 (9%) |
| 4 | 569.362 | 2.178 | 0.177 | H-2→L (19%), H-1→L+1 (66%), H-3→L (2%), H→L+2 (7%) |
| 5 | 550.136 | 2.254 | 0.439 | H→L+2 (89%), H-1→L+1 (7%) |
| 6 | 545.249 | 2.274 | 0.000 | H→L+3 (88%), H-1→L (9%) |

**Table S17:** Wave length, excitation energy and oscillator strength of investigated compound **MR1** in gas phase at B3LYP/6-31G (d, p) level of theory.

| **NO** | **DFT λ (nm)** | **E(eV)** | ***f*** | **MO contributions** |
| --- | --- | --- | --- | --- |
| 1 | 705.337 | 1.758 | 0.219 | H→L (99%) |
| 2 | 697.599 | 1.777 | 0.000 | H→L+1 (99%) |
| 3 | 532.190 | 2.330 | 0.041 | H→L+2 (95%), H-1→L+3 (2%) |
| 4 | 531.505 | 2.333 | 0.000 | H→L+3 (92%), H-1→L (4%), H-1→L+2 (2%) |
| 5 | 512.925 | 2.417 | 0.609 | H-1→L+1 (89%), H-2→L (5%) |
| 6 | 510.097 | 2.431 | 0.000 | H-1→L (90%), H-2→L+1 (3%), H→L+3 (4%) |

**Table S18:** Wave length, excitation energy and oscillator strength of investigated compound **MD2** in gas phase at B3LYP/6-31G (d, p) level of theory.

| **NO** | **DFT λ (nm)** | **E(eV)** | ***f*** | **MO contributions** |
| --- | --- | --- | --- | --- |
| 1 | 776.162 | 1.597 | 0.118 | H→L (98%) |
| 2 | 771.574 | 1.607 | 0.000 | H→L+1 (98%) |
| 3 | 576.858 | 2.149 | 0.000 | H-2→L+1 (13%), H-1→L (72%), H→L+3 (9%) |
| 4 | 576.697 | 2.150 | 0.142 | H-2→L (14%), H-1→L+1 (74%), H→L+2 (6%) |
| 5 | 552.416 | 2.244 | 0.336 | H→L+2 (91%), H-1→L+1 (7%) |
| 6 | 546.885 | 2.267 | 0.000 | H-1→L (11%), H→L+3 (87%) |

**Table S19:** Wave length, excitation energy and oscillator strength of investigated compound **MD3** in gas phase at B3LYP/6-31G (d, p) level of theory.

| **NO** | **DFT λ (nm)** | **E(eV)** | ***f*** | **MO contributions** |
| --- | --- | --- | --- | --- |
| 1 | 900.067 | 1.378 | 0.111 | H→L (99%) |
| 2 | 893.451 | 1.388 | 0.000 | H→L+1 (99%) |
| 3 | 620.107 | 1.999 | 0.000 | H-2→L+1 (11%), H-1→L (79%), H→L+3 (6%) |
| 4 | 619.952 | 2.000 | 0.163 | H-2→L (12%), H-1→L+1 (80%), H→L+2 (3%) |
| 5 | 591.810 | 2.095 | 0.250 | H→L+2 (81%), H→L+4 (12%), H-1→L+1 (5%) |
| 6 | 586.630 | 2.114 | 0.000 | H→L+3 (78%), H→L+5 (11%), H-1→L (9%) |

**Table S20:** Wave length, excitation energy and oscillator strength of investigated compound **MD4** in gas phase at B3LYP/6-31G (d, p) level of theory.

| **NO** | **DFT λ (nm)** | **E(eV)** | ***f*** | **MO contributions** |
| --- | --- | --- | --- | --- |
| 1 | 808.927 | 1.533 | 0.113 | H→L (98%) |
| 2 | 803.787 | 1.543 | 0.000 | H→L+1 (99%) |
| 3 | 586.963 | 2.112 | 0.000 | H-2→L+1 (12%), H-1→L (75%), H→L+3 (8%) |
| 4 | 586.741 | 2.113 | 0.143 | H-2→L (13%), H-1→L+1 (77%), H→L+2 (5%) |
| 5 | 562.389 | 2.205 | 0.300 | H→L+2 (91%), H-1→L+1 (6%) |
| 6 | 556.932 | 2.226 | 0.000 | H-1→L (11%), H→L+3 (87%) |

**Table S21:** Wave length, excitation energy and oscillator strength of investigated compound **MD5** in gas phase at B3LYP/6-31G (d, p) level of theory.

| **NO** | **DFT λ (nm)** | **E(eV)** | ***f*** | **MO contributions** |
| --- | --- | --- | --- | --- |
| 1 | 775.531 | 1.599 | 0.081 | H→L (96%), H→L+1 (2%) |
| 2 | 762.135 | 1.627 | 0.042 | H→L+1 (96%), H→L (2%) |
| 3 | 577.853 | 2.146 | 0.071 | H-2→L (14%), H-1→L (74%), H→L+2 (6%) |
| 4 | 571.646 | 2.169 | 0.081 | H-2→L+1 (13%), H-1→L+1 (74%), H→L+3 (7%) |
| 5 | 550.723 | 2.251 | 0.280 | H→L+2 (83%), H-1→L (6%), H→L+3 (7%) |
| 6 | 542.909 | 2.284 | 0.056 | H→L+3 (82%), H-1→L (2%), H-1→L+1 (8%), H→L+2 (6%) |

**Table S22:** Wave length, excitation energy and oscillator strength of investigated compound **MD6** in gas phase at B3LYP/6-31G (d, p) level of theory.

| **NO** | **DFT λ (nm)** | **E(eV)** | ***f*** | **MO contributions** |
| --- | --- | --- | --- | --- |
| 1 | 877.081 | 1.414 | 0.110 | H→L (99%) |
| 2 | 870.920 | 1.424 | 0.000 | H→L+1 (99%) |
| 3 | 611.845 | 2.026 | 0.000 | H-2→L+1 (12%), H-1→L (78%), H→L+3 (7%) |
| 4 | 611.694 | 2.027 | 0.160 | H-2→L (13%), H-1→L+1 (80%), H→L+2 (3%) |
| 5 | 584.335 | 2.122 | 0.275 | H→L+2 (93%), H-1→L+1 (5%) |
| 6 | 578.662 | 2.143 | 0.000 | H→L+3 (89%), H-1→L (9%) |

**Table S23:** Wave length, excitation energy and oscillator strength of investigated compound **MD7** in gas phase at B3LYP/6-31G (d, p) level of theory.

| **NO** | **DFT λ (nm)** | **E(eV)** | ***f*** | **MO contributions** |
| --- | --- | --- | --- | --- |
| 1 | 698.109 | 1.776 | 0.138 | H→L (98%) |
| 2 | 693.734 | 1.787 | 0.000 | H→L+1 (98%) |
| 3 | 533.426 | 2.324 | 0.000 | H-1→L (42%),H→L+3 (44%), H-2→L+1 (8%), H-1→L+2 (2%) |
| 4 | 533.059 | 2.326 | 0.018 | H-1→L+1(35%), H→L+2 (52%), H-2→L (7%), H-1→L+3 (2%) |
| 5 | 522.435 | 2.373 | 0.475 | H-1→L+1 (49%), H→L+2 (44%), H-2→L (4%) |
| 6 | 517.506 | 2.396 | 0.000 | H-1→L (44%), H→L+3 (52%) |

**Table S24:** Wave length, excitation energy and oscillator strength of investigated compound **MD8** in gas phase at B3LYP/6-31G (d, p) level of theory.

| **NO** | **DFT λ (nm)** | **E(eV)** | ***f*** | **MO contributions** |
| --- | --- | --- | --- | --- |
| 1 | 713.742 | 1.737 | 0.127 | H→L (98%) |
| 2 | 709.942 | 1.746 | 0.000 | H→L+1 (98%) |
| 3 | 551.188 | 2.249 | 0.000 | H-2→L+1 (13%), H-1→L (57%), H→L+3 (24%) |
| 4 | 550.723 | 2.251 | 0.047 | H-2→L (13%), H-1→L+1 (56%), H→L+2 (24%) |
| 5 | 536.612 | 2.311 | 0.456 | H-1→L+1 (23%), H→L+2 (72%) |
| 6 | 530.800 | 2.336 | 0.000 | H-1→L (24%), H→L+3 (72%) |

|  |  |
| --- | --- |
| **MR1** | **MD2** |
|  |  |
| **MD3** | **MD4** |
|  |  |
| **MD5** | **MD6** |
|  |  |
| **MD7** | **MD8** |


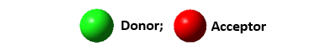


**Figure S1:** The display of Donor and Acceptor structures with green and red colors respectively, in **MR1** and its derivatives (**MD2-MD8**).
